# Development of a neonatal Göttingen Minipig model for dose precision in perinatal asphyxia: technical opportunities, challenges, and potential further steps

**DOI:** 10.3389/fped.2023.1163100

**Published:** 2023-05-04

**Authors:** Marina-Stefania Stroe, Lieselotte Van Bockstal, Allan Valenzuela, Miriam Ayuso, Karen Leys, Pieter Annaert, Sebastien Carpentier, Anne Smits, Karel Allegaert, Adrian Zeltner, Antonius Mulder, Chris Van Ginneken, Steven Van Cruchten

**Affiliations:** ^1^ Comparative Perinatal Development, University of Antwerp, Antwerp, Belgium; ^2^ Drug Delivery and Disposition, KU Leuven, Leuven, Belgium; ^3^ BioNotus GCV, Niel, Belgium; ^4^ Facility for Systems Biology Mass Spectrometry, KU Leuven, Leuven, Belgium; ^5^ Neonatal Intensive Care Unit, University Hospitals Leuven, Leuven, Belgium; ^6^ Department of Development and Regeneration, KU Leuven, Leuven, Belgium; ^7^ Department of Pharmaceutical and Pharmacological Sciences, KU Leuven, Leuven, Belgium; ^8^ Department of Hospital Pharmacy, Erasmus MC, Rotterdam, Netherlands; ^9^ Göttingen Minipigs A/S, Dalmose, Denmark; ^10^ Neonatal Intensive Care Unit, Antwerp University Hospital, Antwerp, Belgium

**Keywords:** animal model, anesthesia, perinatal asphyxia, therapeutic hypothermia, pediatric drug development, precision dosing

## Abstract

Animal models provide useful information on mechanisms in human disease conditions, but also on exploring (patho)physiological factors affecting pharmacokinetics, safety, and efficacy of drugs in development. Also, in pediatric patients, nonclinical data can be critical for better understanding the disease conditions and developing new drug therapies in this age category. For perinatal asphyxia (PA), a condition defined by oxygen deprivation in the perinatal period and possibly resulting in hypoxic ischemic encephalopathy (HIE) or even death, therapeutic hypothermia (TH) together with symptomatic drug therapy, is the standard approach to reduce death and permanent brain damage in these patients. The impact of the systemic hypoxia during PA and/or TH on drug disposition is largely unknown and an animal model can provide useful information on these covariates that cannot be assessed separately in patients. The conventional pig is proven to be a good translational model for PA, but pharmaceutical companies do not use it to develop new drug therapies. As the Göttingen Minipig is the commonly used pig strain in nonclinical drug development, the aim of this project was to develop this animal model for dose precision in PA. This experiment consisted of the instrumentation of 24 healthy male Göttingen Minipigs, within 24 h of partus, weighing approximately 600 g, to allow the mechanical ventilation and the multiple vascular catheters inserted for maintenance infusion, drug administration and blood sampling. After premedication and induction of anesthesia, an experimental protocol of hypoxia was performed, by decreasing the inspiratory oxygen fraction (FiO_2_) at 15%, using nitrogen gas. Blood gas analysis was used as an essential tool to evaluate oxygenation and to determine the duration of the systemic hypoxic insult to approximately 1 h. The human clinical situation was mimicked for the first 24 h after birth in case of PA, by administering four compounds (midazolam, phenobarbital, topiramate and fentanyl), frequently used in a neonatal intensive care unit (NICU). This project aimed to develop the first neonatal Göttingen Minipig model for dose precision in PA, allowing to separately study the effect of systemic hypoxia versus TH on drug disposition. Furthermore, this study showed that several techniques that were thought to be challenging or even impossible in these very small animals, such as endotracheal intubation and catheterization of several veins, are feasible by trained personnel. This is relevant information for laboratories using the neonatal Göttingen Minipig for other disease conditions or drug safety testing.

## 1. Introduction

Pigs are large animal models used in translational research, due to their anatomical, physiological, and biochemical similarities to humans (1, 2). Especially, for drug and food safety testing in the (very young) pediatric population the neonatal pig is considered a good model by regulatory bodies (3–5). Also, for understanding the mechanisms of disease models in neonates, the neonatal pig has been proven to provide useful information. An example is the investigation of the pathophysiology of perinatal asphyxia (PA) in newborn conventional piglets (6–8). PA is a condition defined by oxygen deprivation in the perinatal period, possibly resulting in hypoxic ischemic encephalopathy (HIE) or even death. The neonatal conventional piglet has been very valuable in better understanding the impact and outcome of this condition in patients. However, less is known on the impact of PA versus therapeutic hypothermia (TH), which is the standard approach together with drug therapy to reduce death and permanent brain damage, on drug disposition and thus exposure in these patients. A pig PA model could provide relevant information on the impact of PA and TH on drug disposition, which cannot be assessed separately in patients. However, the conventional pig is not used by pharmaceutical companies for drug discovery or development. The Göttingen Minipig is the commonly used pig strain by the pharmaceutical industry, as they are genetically coherent and well-characterized, and by using the same strain, pharmaceutical companies can rely on historical control data when interpreting findings in drug safety studies (9). Minipigs have already been used as animal models for several clinical indications such as cardiovascular (10), dental conditions (11), diabetes (12), heart disease (13), skin conditions (14, 15), and acute and chronic intestinal inflammation (2, 16). Furthermore, the neonatal Göttingen Minipig shares a striking number of developmental similarities with human neonates (17–21).

As the conventional pig is a well-established model for PA and Göttingen Minipigs are the preferred strain by the pharmaceutical industry, this latter strain provides opportunities for dose precision and potential new drug therapies in PA patients. This project aimed to develop a neonatal Göttingen Minipig model in a neonatal intensive care unit (NICU) setup similar to the human clinical situation, allowing to separately study the effect of the systemic hypoxia versus TH on drug disposition. However, the small size of these neonatal Göttingen Minipigs (∼0.5–0.6 kg compared to conventional pigs, 1.2–1.5 kg) could be challenging for several techniques that are needed in a PA situation, such as endotracheal intubation, vascular access, mechanical ventilation etc.

In this study, we showed that the anatomy and physiology of neonatal Göttingen Minipigs are compatible with mechanical ventilation and multiple vascular access inserted for maintenance infusion, drug administration and blood sampling, in a 24 h non-survival experimental setup, mimicking the clinical situation. Central venous catheterization proved to be the best method for vascular access. Peripheral catheterization was easiest in the epigastric and lateral saphenous veins, whereas catheterization of the umbilical vein depended on whether the umbilical cord was still wet. Finally, in this study we showed that a systemic hypoxic insult could be induced in neonatal Göttingen Minipigs by lowering the inspiratory oxygen fraction (FiO_2_) to 15%, using nitrogen gas. Increased blood lactate and decreased blood pH were used as key parameters for the hypoxia assessment.

## 2. Materials and equipment

### 2.1. Medication

**Figure 1:**
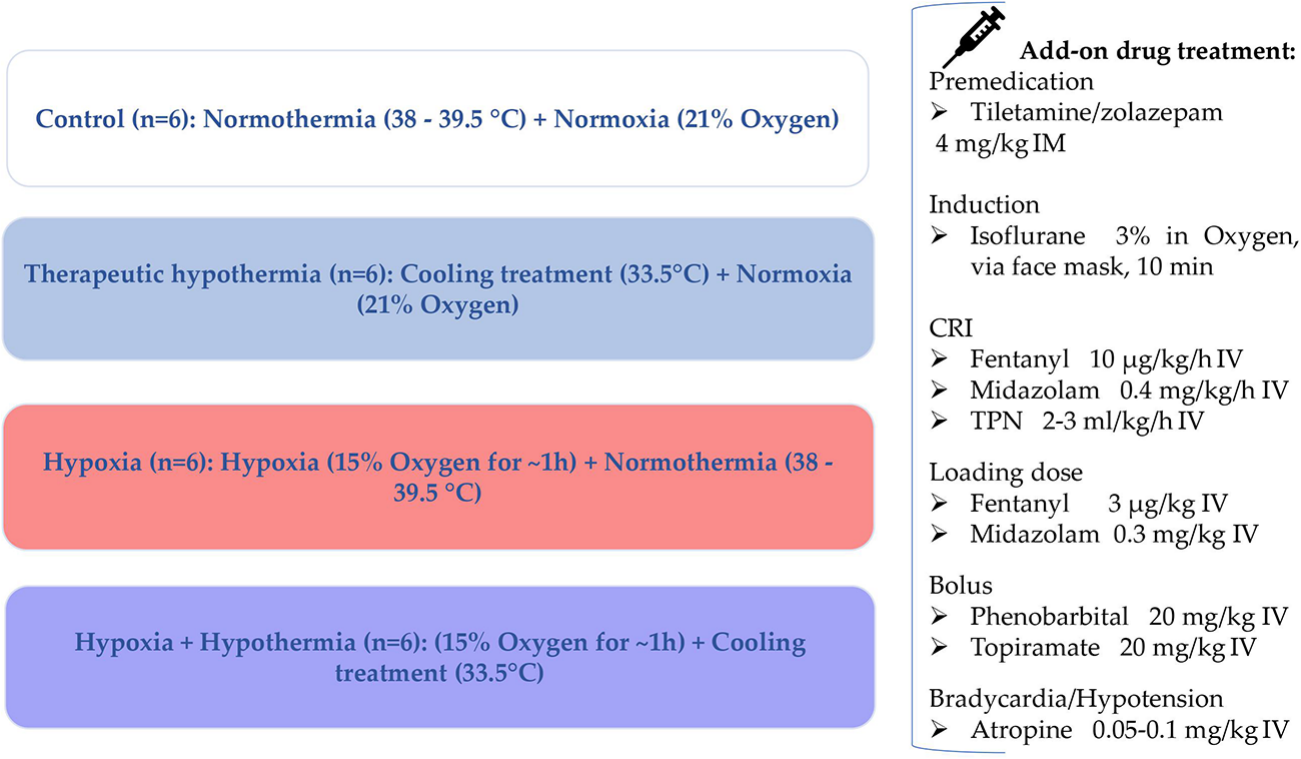
Neonatal Göttingen Minipig groups and the therapeutic interventions. Four conditions, i.e., hypoxia, therapeutic hypothermia, hypoxia + therapeutic hypothermia and controls, with six piglets per condition (*n* = 6), were investigated. °C, degree celsius; h, hour; IM, intramuscular; min, minute; CRI, constant rate infusion; IV, intravenous; TPN, total parenteral nutrition.

In this experimental animal study, the clinical situation of severely asphyxiated neonates was mimicked by administering some of the drugs used in the NICU to the asphyxiated neonatal Göttingen Minipigs during TH. Fentanyl, midazolam, phenobarbital and topiramate were selected as model drugs based on their different physicochemical and/or pharmacokinetic (PK) characteristics and clinical relevance: midazolam (cytochrome P450 (CYP) 3A4; intermediate extraction ratio (ER)), phenobarbital (CYP2C19; low ER), topiramate (largely renally excreted unchanged) and fentanyl (CYP3A4; high ER). The neonatal Göttingen Minipig groups and their therapeutic interventions are depicted in Figure 1. Fentanyl is a full μ opioid agonist, with more profound analgesic effects than morphine (100-fold more potent). The most common indications are pain management and anesthesia. Fentanyl can produce unconsciousness but is accompanied by significant adverse effects such as profound respiratory depression, bradycardia and/or hypotension (22). Midazolam is one of the most used benzodiazepines for premedication and a co-induction agent with ketamine, propofol, or alfaxalone or for maintenance of anesthesia. It acts mainly as a sedative, through a depression of the limbic system, without analgesic properties. Additionally, it is used to manage convulsions, for cases presenting status epilepticus (23). Phenobarbital is a barbiturate used primarily to manage convulsions, without having intrinsic analgesic activity. The anticonvulsive properties of phenobarbital are particularly useful because they tend to provide sufficient motor activity depression, without causing excessive sedation. Liver function is not directly affected when used acutely, but hepatic microsomal enzyme induction is well documented with extended administration of this drug (24). Lastly, topiramate is an anticonvulsant found to be neuroprotectant for hypoxia, ischemia, and convulsions in preclinical models (25). Although topiramate is commonly prescribed for epilepsy and migraine, currently there is no intravenous product available on the market. An intravenous formulation of topiramate has significant advantages over oral formulation since enteral absorption may prove unreliable in critically ill neonates, in which intestinal perfusion and motility are diminished. An injectable topiramate formulation, in which the drug is solubilized in a cyclodextrin matrix, Captisol® (Ligand Pharmaceuticals) has already been developed and proven to be promising for the long-term goal of treating neonatal seizures (25–28).

### 2.2. Monitoring equipment

The heart rate via electrocardiogram (ECG), the fraction of oxygen-saturated hemoglobin (SpO_2_) via pulsoxymeter, end-tidal carbon dioxide (EtCO_2_) and respiratory rate via capnograph, non-invasive blood pressure via blood pressure cuffs (size 1, 3–6 cm, orange fish, 98-0400-80-VET, Suntech Blood Pressure Cuffs), most reliable on radial and medial saphenous arteries, and rectal body temperature were assessed continuously using a multiparameter monitor (MONITOR uMEC12 Vet, Mindray Animal Medical). Mechanical ventilation (ADS 2000 Ventilator Machine), using a pediatric rebreathing circuit, was started immediately after intubation, using a pressure-controlled ventilation mode with a pre-set initial peak inspiratory pressure of 11–15 cm H_2_O, leading to a tidal volume of 10 ml/kg, a respiratory rate varying from 15 to 30 breaths/min, and inspiratory-to-expiratory ratio of 1-to-2 in order to maintain an EtCO_2_ between 35 and 45 mmHg. The most important materials and devices that were required for this PA model development are listed in Table 1.

**Table 1:** List of the most important materials and equipment necessary for the neonatal Göttingen Minipig model development.

Name	Company	Catalog Number	Comments
Doppler Vet BP with 1 probe	-	8289081	–
Blood pressure cuffs	Suntech	98-0400-80-VET	Size 1, 3–6 cm, orange fish
MONITOR uMEC12 Vet	Mindray animal medical	10004054	–
ADS 2000 ventilator machine	Engler engineering corporation	–	–
Uncuffed ETT, mallinckrodt	Covidien	86233	2.5 mm I.D., 3.6 mm O.D.
Hydro-Therm™ Heat and Moisture Exchange (HME) Filter	Intersurgical	1442000	–
BD Microtainer® tube BD Microgard™ closure collections	BD	365974	K2EDTA, closure color lavender
Vamin® 18 gN/L electrolyte-free	Fresenius kabi	065201	–
Glucose 20%	Baxter B.V.	–	–
Intralipid® Caloric Agent Fat Emulsion 20% IV Solution Flexible Bag 500 ml	Fresenius Kabi, baxter B.V	12352211	Lipid Injectable Emulsion, Mfr. Std. Soybean Oil 20%
Vasofix® Safety IV catheter	Braun	4269075	24 G (Yellow)
Venflon™ IV catheter	BD	391451	22 G (Blue)
Discofix® three-way tap	Braun	48919458	–
Original perfusor line	Braun	8722870N	75 cm, 0.8 ml/m
(Arterial) leader catheter	Vygon	115090	3Fr, 8 cm, 24 ml/min
Single-lumen umbilical (arterial) catheter	Vygon	1270.02	PUR, 2.5 Fr, 30 cm, >3 ml/min
Animal polster	Snögg	12116	Foam dressing with adhesive, 9 cm × 2 m × 5 mm
Vicryl 4-0	Ethicon	329-8946	–
i-STAT® Alinity V	Abbott point of care	–	Point of care blood gas analysis device
i-STAT® Alinity V CG4 + cartridge	Abbott point of care	10023271	–
Accutrend® plus system	Roche	4015630056170/5050499171	Lactate meter
Accutrend® lactate strips	Roche	4015630004690	–
Gas Mix (15% oxygen, 85% nitrogen)	Nippon gases, Denmark	-	On special order
WH underbed blanket	OK	OUB 60321	Heating blanket
Contour® XT	Ascensia diabetes care NV/SA	-	Blood glucose monitoring system

## 3. Methods

### 3.1. Experimental design and preanesthetic considerations

**Figure 2:**
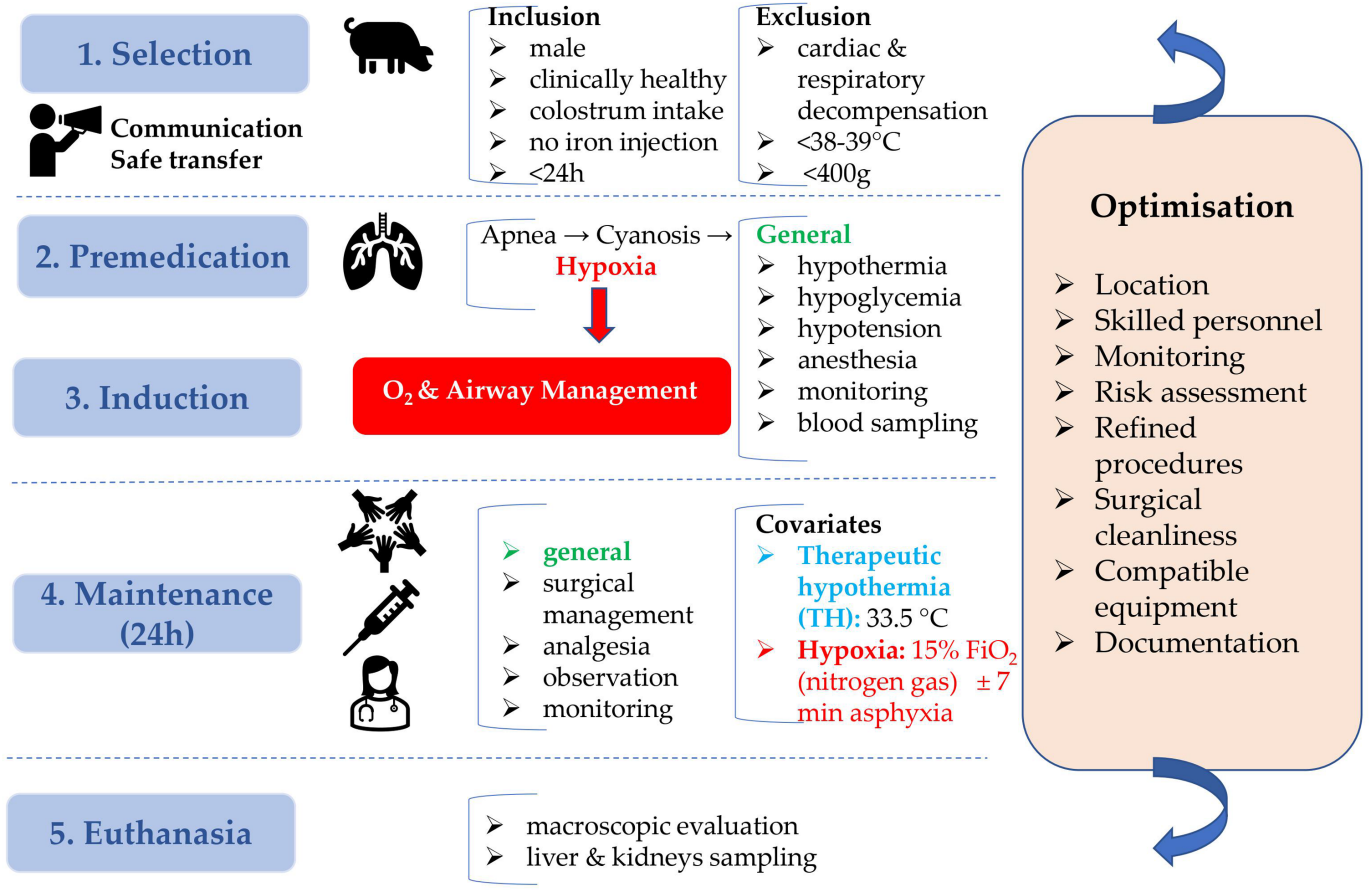
Neonatal Göttingen Minipig study design algorithm. Preanesthetic considerations, with inclusion and exclusion criteria, general risk factors addressed for each study design phase (blue boxes), and optimization (orange box) are depicted. This algorithm emphasizes the importance of planning and preparation in the development of the neonatal Göttingen Minipig model. h, hour; °C, degree celsius; O_2_, oxygen; FiO_2_, inspiratory oxygen fraction; min, minute.

The present animal study was conducted at Göttingen Minipigs A/S, Dalmose, Denmark, under Danish Ethical approval. All subjects were anesthetized throughout the entire procedure. Reproduction of this protocol had to be carried out in accordance with the national ethics and animal welfare guidelines and approved by local ethical committees. Four conditions, i.e., hypoxia (group H), therapeutic hypothermia (group TH), hypoxia + therapeutic hypothermia (group H + TH) and controls (group C), with six piglets per condition, were investigated. A power analysis was performed to determine the correct sample size. This was based on the standard deviation (SD) of dexmedetomidine clearance, from Ezzati et al. (8). Using the clearance as the outcome variable for the four conditions, six piglets per condition are required to detect a 50% reduced clearance, as reported for dexmedetomidine (8), for the H + TH compared to controls (power of 80%; test-wise significance level of 0.05). Further, the procedures within 24 h of experiments are detailed. These were repeated until the achievement of the total number of subjects (24 Göttingen Minipigs) required for the study. While accomplishing prolonged anesthesia, physiological parameters were recorded in individual anesthetic charts, every 10 min. Preanesthetic considerations, general risk factors addressed for each study design phase, and optimization tracks, are presented in a study design algorithm (Figure 2).

Neonatal male Göttingen Minipigs, weighing ∼600 g, coming from different litters were selected in the animal facility at Ellegaard Göttingen Minipigs A/S and immediately transferred to the adjacent operating room. They were clinically healthy, had suckled sufficient colostrum, received no iron injection and were not older than 24 h. At arrival, they were accurately weighed, by using an analytical scale, to allow further precise dosing of the medication. A thorough physical examination was performed to ensure their inclusion in the study. Animals were excluded in case of cardiac or respiratory decompensation at the thoracic auscultation and inspection, signs of hypoxia such as cyanosis, dyspnea with hyperventilation at visual inspection, rectal temperature below the neonatal pig thermal homeostasis threshold (38°C–39°C) (29), congenital anomalies, or in case of a weight below 400 g, considered too small for instrumentation.

### 3.2. Anesthesia

Subjects were premedicated intramuscularly, with tiletamine/zolazepam (Zoletil® 50, Virbac), at 4 mg/kg. Zolazepam is a short-acting benzodiazepine derivative, with tranquilizing properties, available only in combination with tiletamine, a dissociative anesthetic and somatic analgesic (23). This combination is routinely used intramuscularly in various animal species, including pigs, as part of research studies, to induce short-term anesthesia for surgical purposes and for immobilization (30). This preparation was decided for premedication of the neonatal Göttingen Minipigs over alfaxalone (Alfaxan Multidose, Jurox), used at 5 mg/kg. Alfaxalone, a neuroactive steroid molecule (22), has already been documented to produce satisfactory induction and maintenance of anesthesia with minimal cardiovascular side effects in pigs (31). Induction of anesthesia was performed with isoflurane (IsoFlo®, Zoetis) 3% in oxygen, via face mask, for 10 min. If the jaws were relaxed and the palpebral reflex absent, intubation was achieved while placing the piglets in prone position, using uncuffed endotracheal tubes (ETT, Mallinckrodt, Covidien, 2.5 mm I.D. (internal diameter), 3.6 mm O.D. (outer diameter), 86233) and two laces on the upper and inferior jaws. Desensitization with xylocaine spray (Xylocaine® 10%, AstraZeneca) was performed 30 s prior to intubation. Premeasuring and shortening the ETT for an acceptable length, as well as the use of a laryngoscope to visualize and displace the epiglottis from the soft palate, were crucial steps for optimal intubation. The ocular surface was kept moistened by applying eye ointment (Opticorn A, Ecupharm) at frequent intervals. Once intubated, the concentration of isoflurane was gradually decreased, until stopping in the following 1–3 h.

Fentanyl (Fentadon, Dechra), at 10 µg/kg/h, and midazolam (Midazolam, Accord), at 0.4 mg/kg/h, were mixed in one syringe and administered intravenously (IV), at a constant rate infusion (CRI), of 2 ml/h. Additionally, a loading dose of fentanyl, at 3 µg/kg, and midazolam, at 0.3 mg/kg, were administered at the start of the CRI. After 2 h from the start of the infusion and subsequently after 12 h, the other two drugs from the study, phenobarbital (Luminal, Desitin) and topiramate [active ingredient 100%, Cas no 97240-79-4, 339.36 g/mol, Polpharma, solubilized in cyclodextrin matrix, Captisol®, Ligand Pharmaceuticals (25–28)], both at 20 mg/kg, were slowly and separately administered IV, with a 15 min delay in between administrations, to avoid interaction. Total parenteral nutrition (TPN) IV, CRI, followed by close monitoring of glucose status, at the start of the anesthesia and subsequently every 12 h, was performed. The animals received 217 kj/kg/day, representing 50% of the total energetic requirement in neonatal conventional piglets (550 kj/kg/day), from which 8 g/kg/day amino acids (Vamin Fresenius Kabi, Nederland B.V.), glucose 20% (Baxter B.V.) and lipids (INTRALIPID® Fresenius Kabi, Lipid Injectable Emulsion, Mfr. Std. Soybean Oil 20%). It is important to highlight that TPN is recommended to be administered from 6 h after birth and in the first 48 h after starting the solution, just 50% of the total energetic intake is required to achieve normoglycemia (32, 33). The total infusion intake within a day, considering the dilution of drugs and TPN, was 6 ml/kg/h.

### 3.3. Vascular access and body fluids sampling

**Figure 3:**
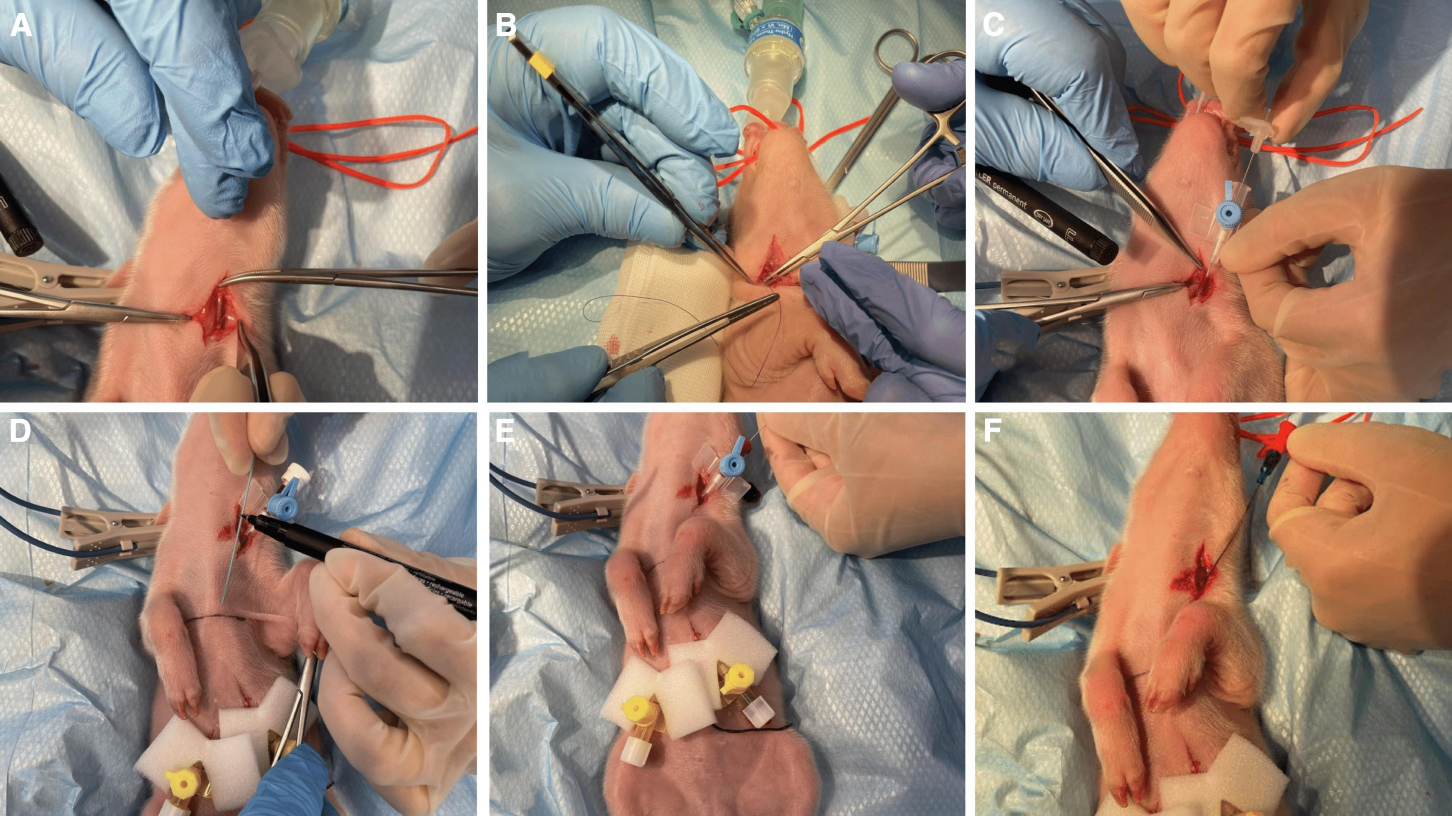
Jugular vein catheterization via modified Seldinger technique in the neonatal Göttingen Minipig (**A**) incision of the skin in the neck region followed by dissection of the jugular vein, (**B**) passing one suture thread under the vein for better manipulation of the vessel and control of the bleeding, (**C**) puncture of the jugular vein with an over-the-needle catheter (BD Venflon™, 22 G, 391451), (**D**) pre-measure the final catheter (Arterial Leader Catheter, Vygon, 3Fr, 8 cm, 24 ml/min, 115090), (**E**) advance the round-tipped guide wire through the lumen of the over-the-needle catheter into the vessel and subsequently withdraw the over-the-needle catheter, (**F**) the final catheter can now be passed over the guide wire and inserted into the vessel to the required length; after that, the guide wire is withdrawn, the catheter is flushed and the skin is closed (Vicryl 4-0, Ethicon). For more stability, one extra suture can be used to restrain the catheter to the surrounding conjunctive tissue before closing the skin.

**Figure 4:**
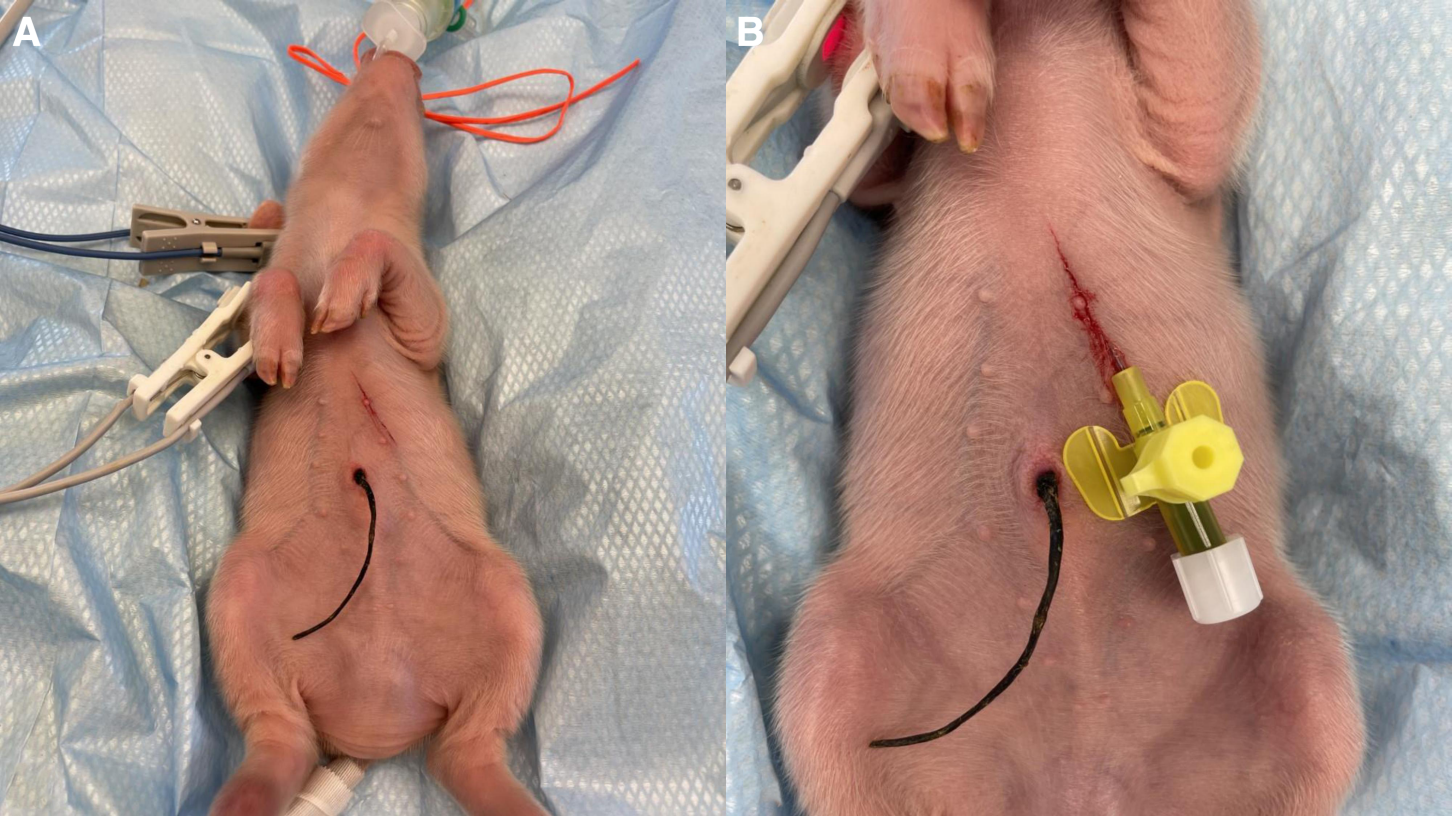
Epigastric vein catheterization: after visualization along the ventral portion of the abdomen and dorsolateral to the mammary chain, and aseptic preparation of the skin, (**A**) a small skin incision was performed before (**B**) catheterization with an over-the-needle catheter (Vasofix® Safety IV Catheter, 24 G, 4269075) followed by confirmation when a (low) blood flow is visible.

**Figure 5:**
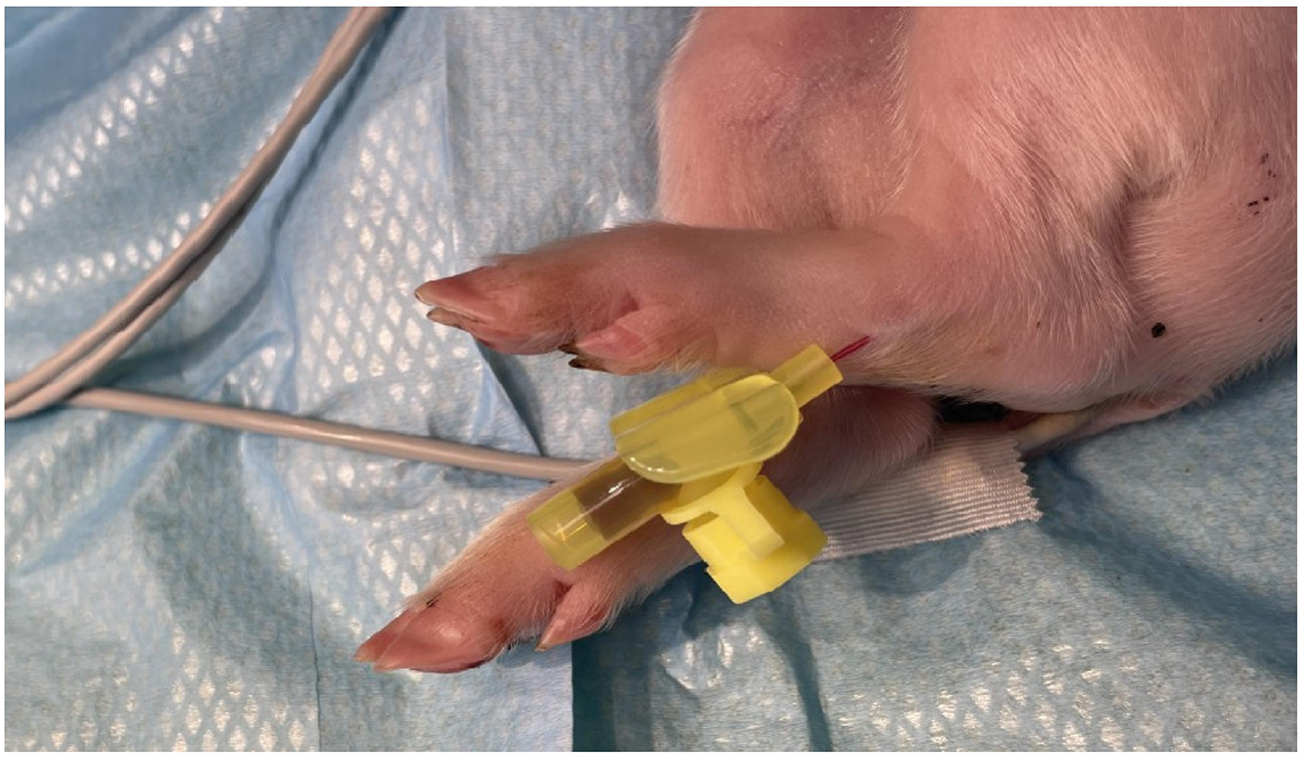
Lateral saphenous vein catheterization with an over-the-needle catheter (Vasofix® Safety IV Catheter, 24 G, 4269075).

**Figure 6:**
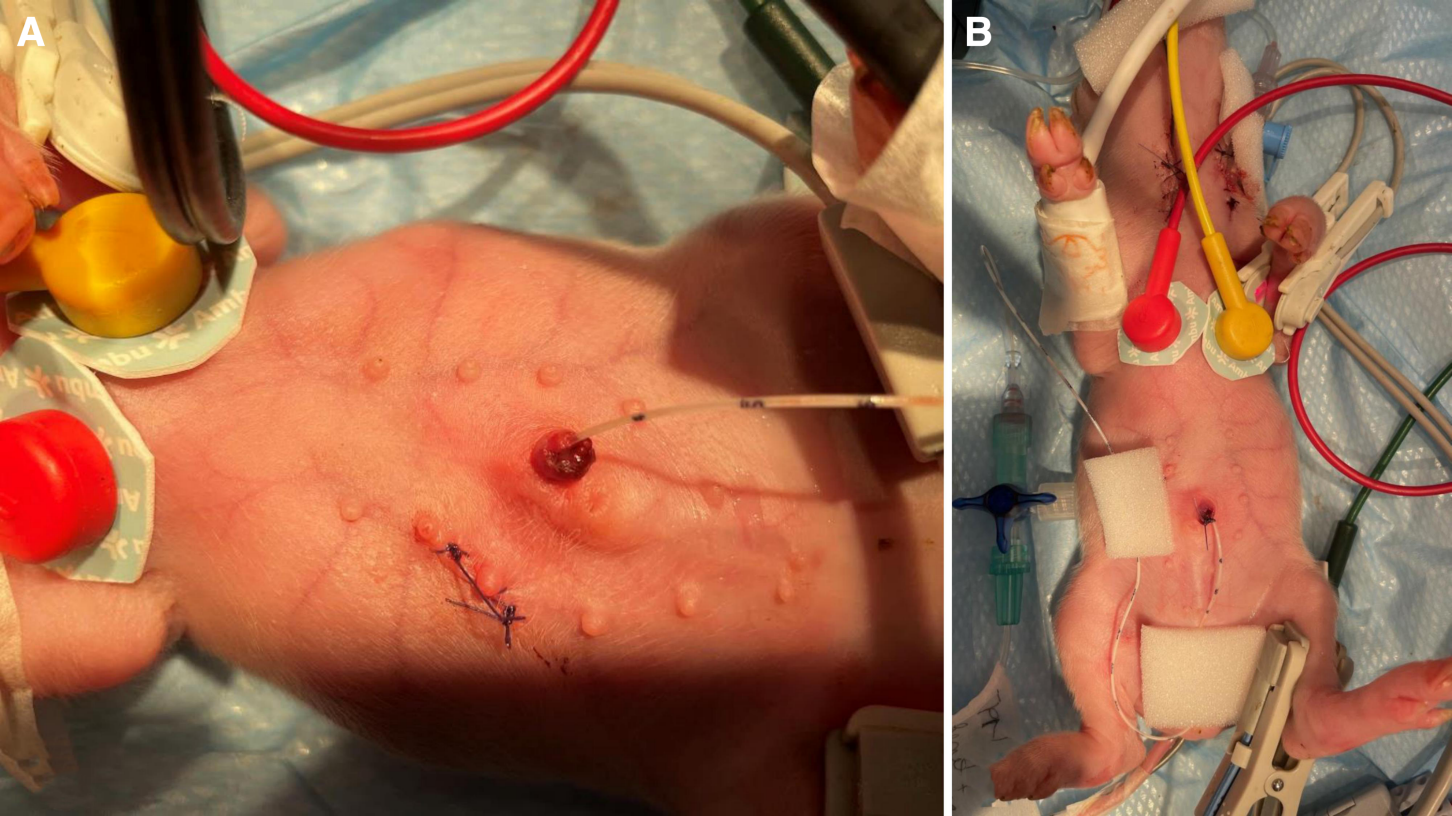
UVC: after cutting the end of the umbilical cord with a scalpel to get a clean surface, leaving an approximate 0.5 cm overhang above the level of the nipple, (**A**) identify the vessels: the vein is large and thin walled, usually at the 12 o’clock position, while arteries are the two thick-walled, below the vein. Grasp the catheter (Vygon, Single-lumen umbilical arterial catheter, PUR, 2.5 Fr, 30 cm, > 3 ml/min, 1270.02) between the thumb and forefinger, or with a forceps, and insert into the lumen of the dilated vein. Place in the inferior vena cava above the level of the diaphragm (between T8 and T9), above the liver. If resistance is met, withdraw slightly, rotate, and reinsert. (**B**) Confirm successful catheterization visually by observing blood return in the catheter or by attaching a syringe and aspirate to see if blood returns and flows easily in the syringe. Tie with suture (Vicryl 4-0, Ethicon) and restrain using the foam dressing with adhesive (Snögg Animal Polster, 9 cm × 2 m × 5 mm) and test the catheter again after fixation by withdrawing blood. Flush the catheter and attach the infusion.

Central venous catheterization, more specifically of the external jugular vein or the junction of external and internal jugular veins (34), was ensured following aseptic procedures, using the modified Seldinger technique (35, 36), while the piglets were positioned in dorsal recumbency. Since the vessels lay deep and are not visible, making the percutaneous technique (37) very challenging, we adapted the approach to a surgical one (Figure 3). To avoid the contamination of the blood sample with the drugs, this catheter was used exclusively for blood sampling. Additionally, for drug administration, two other catheters were placed: either two peripheral catheters or one peripheral and one central. The cranial superficial epigastric vein, also called the milk vein or subcutaneous abdominal vein, was documented for blood collection and fluid delivery in adult conventional pigs (38) and Potbellied pigs (39). The vein courses along the ventral portion of the abdomen and lies dorsolateral to the mammary chain (39). The epigastric vein was visible in the neonatal Göttingen Minipig when placed in dorsal recumbency. An over-the-needle catheter (Figure 4, Vasofix® Safety IV Catheter, 24 G, 4269075) was inserted where the vein is most detectable, in a cranial direction, just lateral to the first and second teat. However, due to the lack of tonicity, a small skin incision was performed before catheterization. The catheter placement was confirmed when a (low) blood flow was visible and was easily flushed. Alternatively, the lateral saphenous vein is a readily accessible vein in pigs (40–42). Blood flows into the caudal femoral vein and then into the external iliac and caudal vena cava (40). This was accessed with an over-the-needle catheter (Figure 5, Vasofix® Safety IV Catheter, 24 G, 4269075) in the neonatal Göttingen Minipigs, placed in lateral recumbency. When the peripheral catheterization was considered challenging, mostly due to poor visualization, the umbilical venous catheter (UVC) was placed. Since this catheter has smaller diameter, a single-lumen umbilical arterial catheter (Vygon, Umbilical arterial catheter PUR, 2.5 Fr, 30 cm, >3 ml/min, 1270.02) proved to be compatible with the neonatal Göttingen Minipigs, this being successfully inserted in the umbilical vein for infusions (Figure 6).

The blood used for PK was limited to 500 µl per sampling and was withdrawn once pre-drug administration and subsequently 10 times after the drug administration, resulting in a total of 11 samples over the study period in each animal. In addition, a heparinized one milliliter syringe was prepared to draw 100 µl blood, followed by quick transfer into CG4 + i-STAT® Alinity V cartridge. Blood gas analysis was performed using the i-STAT® Alinity V. Urine samples were collected via cystocentesis, at the start of anesthesia, at 12 and 24 h. Before cystocentesis, a quick ultrasound check of the urinary bladder was performed. The entire quantity of urine collected over 24 h was recorded.

### 3.4. Hypoxia and therapeutic hypothermia

Using these instrumented Göttingen Minipigs, after stabilization for 30 min and determination of the individual blood gas baseline values, the experimental protocol of normocapnic alveolar hypoxia was started. The piglets were continuously monitored and anesthetized, with fentanyl and midazolam CRI, over the entire period of the insult. Hypoxia was induced by ventilation with a gas mix containing 15% oxygen and 85% nitrogen. During the systemic hypoxic insult, blood gas analysis was performed at 30 min and approximately 1 h after start. Once the blood lactate increased to the threshold of 8.7–10.7 mmol/L and pH decreased from the individual baseline determined before the insult, hypoxia was terminated, at approximately 1 h. When the low oxygen gas mix ventilation was insufficient to achieve the targeted blood gas parameters, occlusion of the ETT was performed for seven min, thus adding hypercapnia to the hypoxia. The insult was followed by reoxygenation with 100% oxygen for 30 min and then 60% oxygen for the remaining time of the experiment. At the end of the hypoxia protocol, phenobarbital and topiramate were administered as mentioned above.

Whole body hypothermia was achieved in less than 90 min using cold packs and by stopping the heating devices, including infrared lamps, and heating mattresses (OUB 60321 WH underbed blanket, OK), and maintained for 24 h, at a target rectal temperature of 33.5°C, in line with the human neonatal target temperature in TH recommendations (43). For the H + TH group, TH was started immediately after finishing the hypoxic insult.

### 3.5. 24 h survival

The experiment was ended after 24 h of drug CRI respectively, after 24 h of hypothermia for the TH and H + TH groups. However, the intended experimental period was 96 h, since TH in the NICU covers 72 h, followed by gradual rewarming and maintenance of 36.5°C for 24 h (43). The rationale for shortening the experimental period to 24 h will be discussed later. The subjects were euthanized while under anesthesia by IV administration of pentobarbital-Natrium (30 mg/kg). Death was confirmed by lack of heartbeat, pulse, corneal reflex, breathing, inability to hear respiratory sounds, and graying of the mucous membranes. After euthanasia, macroscopic evaluation of the abdominal and thoracic cavities was performed and recorded with particular attention to the presence of effusions in the body cavities, macroscopic changes at the level of lungs, stomach, liver, kidneys, intestines, and the placement of the catheters. The liver and kidneys were sampled, divided, snap-frozen, and stored at −80°C for future *in vitro* investigations (e.g., transcriptomics, proteomics).

## 4. Results

### 4.1. Experimental length and subjects

Study procedures were well tolerated in 24 subjects with an average weight of 551.12 (SD ±60.32, minimum 430 and a maximum of 657) g, six for each experimental group and for 24 h. Only one piglet achieved 48 h of anesthesia (605 g, group C), this length being the longest accomplished in the neonatal Göttingen Minipigs reported here. TH was uneventful and easy to control by stopping the heating devices and maintaining it for a total of 24 h, at a target rectal temperature of 33.5°C. For the H + TH group, the duration was prolonged by approximately 2 h since the hypothermia started immediately after finishing the 1-h hypoxic insult and 1-h of instrumentation and stabilization. Comparative overview of the blood gas analysis parameters, glucose, and body temperatures for the (final) neonatal Göttingen Minipigs in function of group: C, TH (end of the experiment), H (end of hypoxic insult), and H + TH (end of hypoxic insult and end of the 24 h TH) groups are depicted in Table 2 (average ± SD in function of the group) and individual parameters (Supplementary Table S1).

**Table 2:** Overview of the (central venous) blood gas analysis, glucose, and body temperature at the moment of the collection (average ± standard deviation).

Parameter	C	TH	H	H + TH	
Time of measurement	24h	24h	End insult	End insult	TH
pH	7.26 ± 0.12	7.1 ± 0.12	6.96 ± 0.08	6.96 ± 0.18	7.164 ± 0.07
PCO_2_ (kPa)	9.82 ± 3.01	14.47 ± 2.93	16.83 ± 0.99	14.43 ± 2.4	13.955 ± 3.08
PO_2_ (kPa)	5.46 ± 1.1	7.01 ± 1.58	2.92 ± 2.31	1.77 ± 0.17	5.5 ± 0.47
HCO_3_ (mmol/L)	32.56 ± 4.1	34.5 ± 18.18	34.1	27.96 ± 3.28	36.36 ± 2.58
BE (mmol/L)	5.5 ± 4.72	6 ± 5.17	4	−2.33 ± 3.78	8.33 ± 2.51
sO_2_ (mmol/L)	64.66 ± 18.57	67.25 ± 35.95	–	7.66 ± 0.57	59.33 ± 7.57
TCO_2_ (mmol/L)	34.83 ± 4.26	37.5 ± 19.75	38	31 ± 3	39.33 ± 2.88
Lactate (mmol/L)	0.97 ± 0.53	0.85 ± 0.8	8.81 ± 2.24	10.13 ± 2.46	0.38 ± 0.06
Glucose (mg/dl)	97.33 ± 23.32	73.16 ± 32.09	80.66 ± 31.94	81.66 ± 19.71	95 ± 16.1
Temperature (°C)	37.7 ± 0.4	34.1 ± 0.95	37.35 ± 1.63	36.01 ± 0.92	33.75 ± 0.75

The assessment was performed at the end of the experiment (24 h) for control (C) and therapeutic hypothermia (TH) groups. For hypoxia (H), and hypoxia and therapeutic hypothermia (H + TH) groups, the parameters were determined at the end of the hypoxic insult and additionally at the end of the 24 h TH. PCO_2_, partial pressure of carbon dioxide; PO_2_, partial pressure of oxygen; HCO_3_, bicarbonate; BE, base excess; sO_2_, oxygen saturation; TCO_2_, total carbon dioxide.

Beside the 24 subjects, two other Göttingen Minipigs accomplished 24 h of anesthesia. They were excluded from the experimental design (group C) since they developed signs of sepsis. The clinical parameters, represented by hypotension [mean arterial blood pressure (MAP) <30 mmHg], tachycardia (>200 beats/min), hypoglycemia (blood glucose level <40 mg/dl), installed gradually, starting at approximately 12 h. The experiment was terminated at 24 h when heart rhythm disorders (extrasystoles) and tachycardia were noticed on the ECG, as well as no response for the low blood glucose interventions and no production of urine. Additionally, low pH (6.87), high partial pressure of carbon dioxide (17.33 kPa) and lactate of 1.04 mmol/L, were diagnostic clues for acidosis detected in one of the piglets. Effusion in the abdominal cavity and macroscopic changes of the lungs, liver, and intestines were noticed at necropsy in both Göttingen Minipigs. The cause of sepsis is not clear, however we suspect a link between pneumonia, respiratory failure, and sepsis, since at that stage of the experimental design, the airway management did not include changeset of recumbency and mouth cleaning on a routine basis. Additionally, we would like to report two other Göttingen Minipigs that achieved 12 h of anesthesia. The first piglet (group C) was intubated with an ETT that had an I.D. of 2.0 mm (Mallinckrodt, Covidien), which was too small, causing air and isoflurane leakage. Due to the inefficient ventilation causing respiratory failure and insufficient anesthesia, this piglet was finally excluded from the study. The second piglet (group H) developed signs of sepsis similar with the ones described above. Slightly white plasma was observed after blood samples centrifugation in this piglet. The cause of the sepsis was confirmed at the necropsy by discovering gastric rupture. Lastly, one neonatal Göttingen Minipig (H + TH3, group H + TH) was excluded from future pharmacokinetic analysis due to epigastric catheter misplacement in the peritoneal cavity, and administration of TPN, phenobarbital and topiramate intraperitoneally. This animal was still used in the assessment of the hypoxic insult, as this technical error did not interfere with the physiology of the animal [see Section 4.4, Supplementary Table S1 (blood gas analysis, glucose, temperature), Supplementary Figure S2 (pharmacodynamic parameters)].

### 4.2. Anesthesia and airway management

Alfaxalone, at 5 mg/kg, and tiletamine/zolazepam, at 4 mg/kg, were the drug candidates tested in the neonatal Göttingen Minipig for premedication. Alfaxalone was well tolerated but provided light sedation and was insufficient for intubation in combination with isoflurane. Subsequently, the combination of tiletamine/zolazepam and isoflurane proved to be more effective and was used for all 24 subjects.

The 2.5 mm I.D. uncuffed ETT was the most adapted in ventilating the neonatal Göttingen Minipigs. This was preferred over a 2.0 mm I.D., which was too small, causing leakage of air and isoflurane, and a 3.0 mm I.D. or cuffed ETTs, which proved to be too big for the trachea. The airway management also included changeset of recumbency and mouth cleaning from mucus and debris using a routine schedule (approximately every 2 h), while ETT suctioning was cautiously used.

All Göttingen Minipigs received TPN with 217 kj/kg/day, from which 8 g/kg/day amino acids were combined with glucose and lipids, administered at 2–3 ml/kg/h. The blood glucose level was checked, using a Contour® XT blood glucose monitoring system, since hypoglycemia represents an anesthesia risk for the neonatal Göttingen Minipigs. In order to assess the blood glucose status in the neonatal Göttingen Minipigs investigated, thresholds previously described as mild hypoglycemia (60 mg/dl) and moderate hypoglycemia (35 mg/dl) in the neonatal conventional piglets, were considered. This TPN formulation was sufficient to maintain normoglycemia over the entire length of experiment. Consequently, the average blood glucose level before drug administration was 91.95 (SD ±25.60, minimum 42 and a maximum of 134) mg/dl, after 12 h of anesthesia 86.42 (SD ±18.54, minimum 57 and a maximum of 128) mg/dl and at the end of the experiment 87.31 (SD ±25.65, minimum 30 and a maximum of 133) mg/dl.

### 4.3. Vascular access

Three different venous catheters were placed in each neonatal Göttingen Minipig of the 24 investigated here, as follows: 43 central catheters, including 38 jugular catheters and 5 UVCs, as well as 29 peripheral catheters, including 26 epigastric and three lateral saphenous. Central venous catheterization, via the modified Seldinger technique, showed to be the primary method for vascular access, either for sampling or drug administration, in the neonatal Göttingen Minipig. This method possessed internal consistency and reproducibility, since it was performed multiple times by the assessor and the technician, throughout the study. Specifically in our study, the peripheral catheterization was most feasible in the epigastric and the lateral saphenous veins. The auricular vein was considered too small in neonatal Göttingen Minipigs. Some neonatal Göttingen Minipigs were more difficult to catheterize on the peripheral vessels than others. Therefore, the catheterization of the umbilical vein was performed using the single-lumen umbilical arterial catheter (Vygon, Umbilical arterial catheter PUR, 2.5 Fr, 30 cm, >3 ml/min, 1270.02). The success of this technique depended on the sufficient moisturizing of the umbilical cord after birth. Blood sampling was performed for PK, which will be performed and discussed in a later phase of the project, as well as for blood gas analysis. This was uneventful and easy to perform via the jugular catheters.

### 4.4. Hypoxia

Hypoxia could be established for an average of 51 (SD ±34.82, minimum 20 and a maximum of 145) min in neonatal Göttingen Minipigs. This was possible by ventilating with a gas mix containing 15% oxygen and 85% nitrogen, or by combining the low oxygen gas mixture with asphyxia, performing the ETT occlusion, for seven min. The body weight, the hypoxia length, and the need for the ETT occlusion to induce the insult are depicted in Table 3. This degree of oxygen deprivation was sufficient to produce hypoxia with cyanosis, a drop of SpO_2_ at approximately 40%–20%, or tachycardic response (>200 beats/min) followed by severe bradycardia (<75 beats/min), with severe acidosis, cardiogenic shock and hypoperfusion to vital organs. Atropine, at 0.05–0.1 mg/kg, was used to correct severe bradycardia and hypotension in seven neonatal Göttingen Minipigs. The blood gas analysis, glucose, and body temperature for each neonatal Göttingen Minipig are provided as supplementary materials (Supplementary Table S1), as well the trend lines for the key pharmacodynamic parameters in assessment of the systemic hypoxic insult (Supplementary Figure S2). Graphs were made in Microsoft Excel® version 16.0.1, 2021 (Microsoft Corporation, Redmond, WA, United States).

**Table 3:** The body weight, the hypoxia length, and the need for the ETT occlusion to induce the insult in neonatal Göttingen Minipigs.

	Animal	Group	Weight (g)	Length of hypoxia (min)	Endotracheal tube occlusion
1	H1	H	521	20	–
2	H2	H	502	38	–
3	H3	H	430	35	–
4	H4	H	531	30	–
5	H5	H	547	20	Yes
6	H6	H	592	45	–
7	H + TH1	H + TH	530	45	–
8	H + TH2	H + TH	610	145	Yes
9	H + TH3 (Note 1)	H + TH	540	50	–
10	H + TH4	H + TH	507	50	–
11	H + TH5	H + TH	552	55	–
12	H + TH6	H + TH	650	30	–
13	H + TH7	H + TH	521	100	Yes

H, group hypoxia; H + TH, group hypoxia + therapeutic hypothermia; g, grams, min, minute. Note 1: For H + TH3 the systemic hypoxic insult and 24 h survival was achieved. However, at the end of the experiment, misplacement of the epigastric catheter was noticed. Consequently, this subject was excluded from the pharmacokinetic analysis. Since this animal was excluded due to a technical and not a physiological issue, its blood gas analysis was still included in the hypoxia statistical analysis.

**Figure 7:**
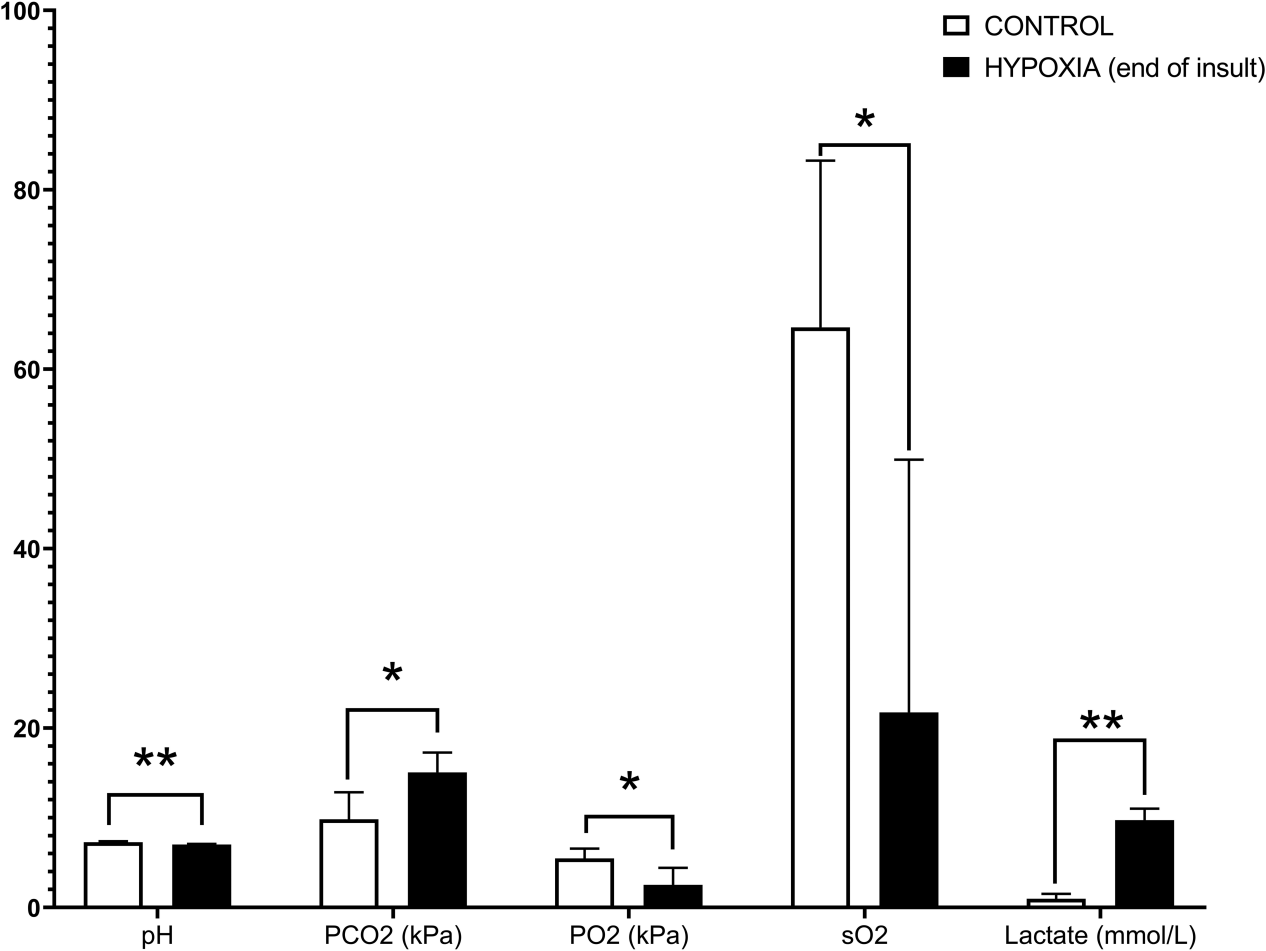
Hypoxia-related differences within the controls and the hypoxic groups for blood gas analysis. A *p*-value smaller than 0.05 was considered statistically significant. Values were statistically significantly higher for PCO_2_ (partial pressure of carbon dioxide, *p*-value: 0.0073) and lactate (*p*-value: 0.0039), while for pH (*p*-value: 0.0032), PO_2_ (partial pressure of oxygen; *p*-value: 0.037), sO_2_ (oxygen saturation; *p*-value: 0.0325) were statistically significantly lower. Designed in GraphPad Prism version 8.0.2 (GraphPad Software, Inc. San Diego, California USA); *p*-value: *, *p* < 0.05; **, *p* < 0.005; ***, *p* < 0.0005; ****, *p* < 0.0001.

An increase in blood lactate (average 9.56, SD ±2.27 mmol/L) and a decrease in blood pH (average 7.00, SD ±0.16) were used as biomarkers for the systemic hypoxic insult. A high lactate level is a diagnostic clue for tissue oxygen deprivation, indicating the switch from aerobic metabolism to anaerobic. A comparative overview of the average ± SD of the blood gas analysis parameters for C, TH (end of the experiment), H (end of hypoxic insult), and H + TH (end of hypoxic insult and end of the 24 h TH) groups are depicted in Table 2. The statistical analysis was performed in JMP® Pro 16 (SAS Institute Inc., Cary, NC, United States). Normality and homogeneity of variances were tested by the Shapiro-Wilk and Levene's test, respectively. The Student *t*-test was used to detect hypoxia-related differences between the controls and the hypoxic groups and for each blood gas parameter. If assumptions could not be met, a non-parametric Wilcoxon/Kruskal-Wallis (rank sums) test was performed. A *p*-value smaller than 0.05 was considered statistically significant. Values were statistically significantly higher for PCO_2_ (partial pressure of carbon dioxide, *p*-value: 0.0073) and lactate (*p*-value: 0.0039) while for pH (*p*-value: 0.0032), PO_2_ (partial pressure of oxygen; *p*-value: 0.037), sO_2_ (oxygen saturation; *p*-value: 0.0325) were statistically significantly lower (Figure 7).

## 5. Discussion

In this study, we developed a neonatal Göttingen Minipig model for dose precision in PA. The need for this nonclinical model refers to the clinical context, where the impact of systemic hypoxia and TH on drug disposition cannot be studied separately. Different techniques and protocol steps were performed to establish this neonatal animal model. The critical decisions, as well as the challenges, limitations and potential further steps, will be further discussed.

### 5.1. Airway management

Due to the unique laryngeal anatomy and their predisposition for laryngospasm, endotracheal intubation is demanding in pigs (44, 45). Intubation is needed in a PA piglet study design since the tracheal tube maintains a patent airway during general anesthesia, protects the airways from aspiration, and allows intermittent positive pressure ventilation (IPPV) (46). Nevertheless, the piglet's anatomical design does not facilitate intubation, as it has a narrow glottis and trachea relative to overall animal size. The long soft palate, fatty cheeks, jowls and tongue, and narrow jaw angle also make the visualization of the epiglottis difficult. Both dorsal and ventral recumbency are the alternatives most frequently described for endotracheal intubation in pigs. Ventral recumbency, the method adopted in our study, enables smooth and fast intubation and reduces the risk of airway obstruction (45). However, if the head is overextended, it increases the risk of laryngeal obstruction. Interestingly, dorsal recumbency usually allows the excessive pharyngeal tissue to “fall away” from the glottis, thus providing a good airway visualization (47). In our study, the first piglet was intubated with an ETT that had an I.D. of 2.0 mm (Mallinckrodt, Covidien), which was too small, causing air and isoflurane leakage. Due to the inefficient ventilation and insufficient anesthesia this first piglet, destined to the control group, was finally excluded from the study. Finally, the 2.5 mm I.D., 3.6 mm O.D. uncuffed ETT (Mallinckrodt, Covidien, 86233) was the most adapted in ventilating neonatal Göttingen Minipigs. Particularly, this issue is also encountered when ventilating human newborns. Neonates are commonly ventilated using uncuffed endotracheal tubes. This may lead to a variable leakage around the ETT, depending on spontaneous breathing, the inspiratory pressure used, lung compliance, neck position and position of the ETT itself. Therefore, if the delivered tidal volume is measured only at inspiration, there can be a large discrepancy between pre-set and delivered volume, by overestimation (48). ETT patency is important for mechanically ventilated patients. Particularly in the NICU, for patients in whom small-sized ETTs are used, the risk of occlusion by secretions and subsequent need for reintubation may be high. Therefore, tube suction might be critical in order to avoid obstruction from mucus and debris. On the other hand, ETT suctioning is associated with many well-documented deleterious effects such as: bradycardia, desaturation, atelectasis, increased intracranial pressure, bronchial perforation/pneumothorax. Reducing the incidence of these side effects is inherent in the method in which ETT suctioning is performed. Limited frequency, shallow suctioning and preoxygenation are some beneficial practices (49). For the airway management of the neonatal Göttingen Minipigs reported here, the mouth was cleaned approximately every 2 h from mucus and debris together with changeset of recumbency while ETT suctioning was cautiously used. The ETT suction procedure was needed when visible secretions in the ETT or when changes in vital signs were noted, when the amplitude of the chest inflation at inspiration decreased, or abnormal breath sounds (i.e., wheezing) were noted. Interestingly, it was noted that the frequency of ETT suction and the reintubation need decreased when a predefined schedule of recumbency change was set up. The piglets could experience arterial oxygen desaturation very rapidly during multiple steps throughout the protocol, especially during different manipulations for airway management and intubation (44). Oxygen supplementation at 100%, 3 L/min was immediately provided via face mask or ETT until the restoration of SpO_2_.

Pigs, like other mammals, are prone to respiratory depression under general anesthesia. Additionally, they are prone to suffer from partial airway obstruction, due to the species-specific reduced functional residual capacity of the lungs and the presence of excess tissue, in the oropharyngeal region. This increases the likelihood of peri-anesthetic hypoxia (46). Furthermore, besides respiratory fatigue in prolonged anesthesia, during spontaneous breathing, dependent lungs, understanding the lowest part of the lung in relation to gravity, tend to collapse if not periodically inflated. Atelectasis prevails in dependent healthy lungs under general anesthesia. It is a position and pressure-dependent phenomenon and no matter in which position the patient is placed, the gravity-dependent lung zones will always be susceptible to airway closure and atelectasis (50). Areas of diffuse atelectasis will fail to oxygenate the blood. Therefore, venous admixture will lower blood oxygen levels (51). To prevent this, mechanical ventilation, by imposing a positive inspiratory pressure at a fixed frequency, maintained stable blood gas values, and preserved metabolic energy. IPPV was the choice in our study, together with the frequent changeset of recumbency to further prevent atelectasis. On the other hand, there is also a continuous background pressure, known as positive end expiratory pressure (PEEP, baseline pressure maintained during expiration), and this is an important ventilator setting to stop lungs from collapsing especially in human neonates (52, 53). This setting was more challenging to achieve in our neonatal Göttingen Minipigs due to their smaller size. Specific neonatal ventilators are recommended for further prolonged anesthesia studies using the neonatal Göttingen Minipig as model. Inadequate mode of ventilation for healthy lungs, can cause significant changes in their structure and function leading to ventilator induced lung injury (VILI) (54). This can be particularly encountered in mechanically ventilated small animals and potentially result in excessive pressures (barotrauma), excessive distending volumes (volutrauma), alveolar damage resulting from transient and repeated closure and reopening of alveoli during the respiratory cycle (atelectotrauma), and biotrauma, in which the altered magnitude and pattern of lung stretch changes gene expression and cellular metabolism in a way that produces an overwhelming inflammatory response (51). On the opposite side, small tidal volumes will produce an insufficient exchange of alveolar gases, no matter the respiratory rate or minute volume. This, in turn, would rapidly lead to carbon dioxide retention and the expected complications of hypercarbia and respiratory acidosis. Additionally, progressive atelectasis, deteriorating ventilation-perfusion and impaired oxygenation will eventually happen (48).

### 5.2. Vascular access

The main reason for using a venous catheter is to facilitate multiple blood sampling or intravenous dosing. It reduces animal stress, improves welfare, and reduces the number of personnel required (34). Prompt placement of a peripheral intravenous catheter allows treatment of emergencies if they arise during induction. On the basis of this experimental study results, we recommend the use of the epigastric vein for IV administration of fluids and medications in neonatal Göttingen Minipigs. The piglets reported here were remarkably tolerant of this catheterization procedure and subsequent drugs and fluid administration. Catheter placement was successful when a small incision of the skin was performed before catheterization. We did not detect any unfavorable sequelae to the catheterization or drugs and fluid administration, such as infection and thrombophlebitis. As a limitation, the use of the epigastric vein for blood collection may prove to be inferior to other means of collection (i.e., central venous catheterization) because of the relatively low blood flow. Additionally, there is a risk of inadvertently entering the peritoneal cavity during attempted venipuncture by directing the stylet of the over-the-needle catheter too deeply. This last complication was detected in one neonatal Göttingen Minipig that was finally excluded from the study. Alternatively, the lateral saphenous vein was an appropriate method for IV administration of fluids and medications in neonatal Göttingen Minipigs. However, compared with the epigastric vein, the catheterization of the lateral saphenous vein was slightly more difficult. This vein is embedded within subcutaneous fat and fascia and is not immediately apparent in pigs. It was already reported that incision of the skin above the hock, below the fleshy portion of the leg, and more proximal to the Achilles tendon increase the ease of canulating this vessel (40).

Although peripheral veins were used successfully in several neonatal Göttingen Minipigs reported here, some piglets were more difficult to catheterize than others. When talking about very small subjects the target vessels for blood collection and IV administration of fluids and medications are the central ones. Therefore, the central venous catheterization was the preferred method for vascular access in neonatal Göttingen Minipigs. The most common site is the neck, where the external jugular vein or the junction of external and internal jugular veins is catheterized (34). In prolonged procedures, it is necessary to ensure a high level of surgical cleanliness when cannulating blood vessels and performing other “routine” instrumenting procedures. Infection becomes more likely the longer catheters are in place (51). The UVC is one of the most commonly used central lines in human neonates. It can be easily inserted soon after birth providing stable intravenous access for resuscitation medication, fluids, and parenteral nutrition during the first days of life (55). As with other central catheters, thromboembolic events, bacteremia, and sepsis are common complications of UVC use. Complications that are particular to UVCs commonly are the result of the catheter malposition in the heart, more specifically in the atria, resulting in cardiac arrhythmias, or in the portal system, resulting in serious hepatic injury. Theoretically, a catheter that is placed beyond the right atrium may enter the superior vena cava or the right ventricle, although in reality, most often this catheter may pass through the “foramen ovale” into the left atrium. From there, the catheter may enter the left ventricle or a pulmonary vein. In these cases, the catheter must be withdrawn from the heart and returned to the inferior vena cava. On the other hand, hepatic necrosis can occur due to thrombosis of the hepatic veins or intravascular catheter complications, infusion of hypertonic or vasospastic solutions into the liver. Necrotizing enterocolitis and perforation of the colon and/or peritoneum have been reported following the positioning of the catheter in the portal system (56).

### 5.3. Blood sampling

Piglets are generally injected intramuscularly with iron (2 or 3 days) after birth to prevent anemia and improve growth (57, 58). We observed that iron injection has an impact on the collected samples by coloring the plasma and other organs, thereby potentially influencing further *in vitro* investigations (e.g., transcriptomics). This learning was aligned with the literature findings since it was documented that iron injections increas the hemoglobin concentration (57). Additionally, hemoglobin in whole blood can lead to clogging of extraction matrices, which makes blood RNA isolation further difficult and gene expression analyses unreliable (59). On the other hand, piglets are born with very low iron stores and potentially become iron deficient. However, this might happen right before weaning (approximately 20 days old) (58). The neonatal Göttingen Minipigs recruited for our study did not receive iron supplementation since the follow-up of pigs after birth was short (limited to 24 h), as well as to prevent compromising future *in vitro* investigations. Blood sampling was uneventful and easy to perform via central venous access. One limitation in the view of the blood sampling and the size of the model should be emphasized. As PK studies are demanding in terms of blood samples (preferably requiring rich data sets), the animal size and therefore volume of blood, that can be collected over 24 h, represents a limitation. In our study design, this was accomplished, via micro sampling, the amount of blood collected was below 15% of the piglet's total blood volume, counting the samples for PK, blood gas analysis, and the possible blood loss during catheter placement. Since the sampling covered 24 h while piglets received an IV infusion, no signs of cardiac decompensation were noticed. However, this degree of blood loss is sufficient to induce hypovolemia in certain individuals, especially when the collection is performed intensively at certain time periods. Therefore, for further perspectives in PK studies using the neonatal Göttingen Minipig as model, the volumes of blood collected should be cautiously determined, both from an ethical and study design validity points of view.

### 5.4. Prolonged anesthesia

Monitoring of non-invasive vital signs should begin as soon as the animals are included in the study. The plane of anesthesia, MAP, heart rate, SpO_2_, body temperature and EtCO_2_ can be readily obtained and monitored (51). This is critical for the early detection of potential problems during the entire anesthetic period. In the perioperative mortality study by Brodbelt (60), many factors significantly contributed to mortality but through monitoring the risk of death was significantly decreased (22, 60). One of the contributing factors in anesthesia-related death is hypothermia (60). Neonatal and pediatric animals are highly susceptible to hypothermia because of their high body surface area-to-body mass ratio. As these animals can become quickly hypothermic under general anesthesia, the management should include numerous strategies (51): creating warm “local” conditions (bubble wrap insulation, with hot air blankets and/or heater pads), minimalization of the heat loss using IPPV judiciously, favoring “low-flow” re-breathing systems and using heat moisture exchangers. However, these heat losses, promoted by surgery and anesthesia, proved to be useful in our study, for the hypothermia groups, by inducing gradual cooling, over 90 min, in a less expensive setup than the clinical one (e.g., MiraCradle-Neonate Cooler).

Prolonged anesthesia may be required in studies using pigs as models for the human intensive care patient. In these type of studies, it is difficult to achieve a balance between prolonged anesthesia to maximize the data set and maintain a stable physiological state, and to minimize data variability and increase the study power (51). Particularly, one limitation of our model refers to the length of anesthesia. The initial target period was 96 h, since TH in the human setting covers 72 h, followed by gradual rewarming and subsequently maintenance of a body temperature at 36.5°C during 24 h (9). However, after several attempts the length of the experiment was reduced to 24 h and the dose regimen and sampling scheme were adapted accordingly. The rationale first of all relates to the complexity of the animal model with the logistical challenges. Furthermore, there is also the need for skilled investigators in permanence for the entire length of the experiment, as well as a fully supplied operating suite, comparable to the human NICU, which is more sophisticated than the typical veterinary operating suite. The techniques in developing piglets for experimentation require experience to master, for the investigator and his assistant, and they must be comfortable with both the surgery and anesthesia. This was achieved by performing a preliminary pilot study in which we developed the PA model using conventional piglets to refine the techniques, and to optimize the experimental setup. The sample size of this pilot study was determined by the experience and the personal judgment of the investigator and was limited to eight animals. As a consequence of the 24 h limitation, the rewarming was skipped in this study design, but it opened the possibility for further research directions.

Adjustments of anesthesia were performed to maintain homeostasis. Hypotension, hypoglycemia, shivering during TH, transitory insufficient anesthesia, and ventilation troubleshooting, such as carbon dioxide retention, high production of mucus, ETT leak, and lung atelectasis, are the main difficulties that we countered through comprehensive monitoring and customized procedures, as necessary. However, appropriate anesthetic management of neonatal and pediatric animals is often differently compared to adults. Clinically acceptable ranges can be different in neonates compared to adults; therefore the anesthesiologist should be familiar with the acceptable ranges for the species and age group of individuals that are being anesthetized (22). Generally, neonatal, and juvenile animals have a higher heart rate but lower blood pressure than adults. For example, 45 mmHg represents the lower limit of autoregulation in neonatal swine for the MAP (61, 62), while 117.8 (in mean with SD ±4.7) mmHg was the reported MAP in freely moving adult Göttingen Minipigs (63). Compared with the adult heart, the neonatal heart has limited myocardial contractile tissue, low ventricular compliance, limited cardiac reserve, cardiac output heart rate dependent and poor vasomotor control. The cardiovascular system must be supported with IV fluids and chronotropic support. Anticholinergic drugs are commonly used to treat and/or prevent anesthetic and preanesthetic bradycardia, decrease airway and salivary secretions, dilate the pupil, block vagally mediated reflexes (viscerovagal, oculocardiac, Branham), and block the effects of parasympathomimetic drugs (22). In our study, atropine at 0.05–0.1 mg/kg was well tolerated by the neonatal Göttingen Minipigs. It represented a valuable drug used in stabilizing periods, after induction or hypoxic insult, when bradycardia and hypotension occurred. Since hypoglycemia is one of the most common neonatal anesthesia risks, TPN IV, CRI, followed by close monitoring of glucose status, every 12 h, was performed. The TPN formulation for neonatal piglets consisted of 3,200–3,400 kj/L including: amino acids (45.5–54 g/L), glucose (72.5–116 g/L) and lipids (21–30.7 g/L) (32, 33). For the neonatal Göttingen Minipigs reported here, the TPN solution was administered continuously via the UVC or the peripheral catheters. The piglets received 217 kj/kg/day, from which 8 g/kg/day of amino acids were combined with glucose and lipids. This represents 50% of the total intake in neonatal piglets (550 kj/kg/day) and the energetic requirement necessary for the first 48 h. Because of the high osmolarity of the TPN, supplemental crystalloids (e.g., 0.9% NaCl) were administered to maintain fluid homeostasis. Shivering, caused by light anesthesia and hypothermia, increases oxygen consumption (up to 200%–300%). This increased oxygen demand may not be met by an increase in oxygen delivery, particularly if anesthetic-induced hypoventilation occurs (22). The light anesthesia was addressed by starting the CRI of fentanyl and midazolam with loading doses, allowing the smooth change from gas anesthesia to total intravenous anesthesia (TIVA).

### 5.5. Potential further steps

The aim of this experimental study was to develop a neonatal *in vivo* model to assess the impact of systemic hypoxia and TH on drug disposition, to support improved drug dosing and drug development in asphyxiated neonates. As the potential role of hypoxia and TH cannot be explored separately in the clinical situation, this model is of utmost importance since it allows this distinction. The I-PREDICT project (Innovative physiology-based pharmacokinetic model to predict drug exposure in neonates undergoing cooling therapy) mainly focuses on drug metabolism and elimination, targeting the liver and the kidneys. However, regarding investigations, the limitation referring to the examination of other organs (i.e., brain) and systems that were not performed in this study needs to be emphasized. We developed a novel animal model for drug disposition and not an animal model for HIE (and thus not to assess brain damage). The construct validity of this model was based on blood lactate and pH, biomarkers in the determination of systemic hypoxia and not necessary on neuropathological assessment, since HIE was out of our scope. Therefore, when considering our model for HIE research, additional diagnostic tools are needed to assess its validity. Some of them are discussed below.

#### 5.5.1. Additional diagnostic modalities for perinatal asphyxia and hypoxic ischemic encephalopathy

In the NICU, the diagnosis of PA is based on clinical endpoints [Apgar score ≤5 at 10 min after birth (9, 64)], failure to initiate or sustain spontaneous breathing at birth (65) leading to impaired gas exchange (66), clinical chemistry results, such as metabolic acidosis identified via the arterial umbilical cord blood obtained at delivery (67). For diagnosing HIE, clinical scoring or more sophisticated tools like electroencephalography (EEG) are used. In addition, neuroimaging is used to evaluate the impact of asphyxia on brain structures (see Section 5.5.1.1) (65, 68). Also, the pathological diagnosis of PA is important, which is based on histological markers of tissue hypoxia, with a relevant role in vascular changes (69). All these diagnostic parameters could prove useful as well when performing a research study. These will provide important evidence for better understanding the mechanisms of PA and confirming the degree of hypoxia. However, the selection of the parameters and the interpretation of the results should be carefully carried out. Therefore, when designing a PA/HIE animal study several considerations need to be taken into account: species-tailored clinical diagnostic, the maturational stage of the brain at the time of injury which differs from species to species (70), the use of anesthesia [which may alter the neurological examination (65)], but also the neuroprotection protocols (TH, medication), the special training for more sophisticated techniques, the presence of a multi-disciplinary team to perform full monitoring and critical care, and the costs and logistics for making all these techniques available in the research setup. These diagnostic modalities have already been used in PA/HIE translational research, using the conventional pig model (7, 71, 72). While it should be technically feasible to extrapolate these modalities to the minipig, this necessitates further studies.

##### 5.5.1.1. Neurological assessment

Amplitude-integrated electroencephalography (aEEG) is a technique for simplified EEG, which is applied in the NICU. Its main value lies in allowing to monitor baseline brain activity, and real-time detection of electrographic seizures, providing the opportunity for treatment at the time they occur, without requiring extensive formal training for their interpretation (73). To avoid artefacts misdiagnosed as seizures, the aEEG trace preferably must be reviewed by conventional, full, EEG. However, the interpretation of conventional EEG requires more specific expertise (73). Overall, neurological assessment (clinical as well as by EEG) to diagnose HIE, is ideally performed before the administration of sedatives that may alter the neurological examination. The final (a)EEG pattern is indeed a combined reflection of the disease state, medication administered and the impact of TH [potential accumulation of sedatives, affecting the (a)EEG (74)]. Previous preclinical studies with the pig model have shown that a more consistent brain injury can be achieved, with an enhanced survival rate, by individualizing the hypoxic insult according to each subject's cerebral response evaluated by aEEG. Models of global hypoxia-ischemia titrated by aEEG suppression (aEEG flat trace, upper margin <7 µV), have demonstrated encephalopathy that is clinically, and neuropathologically comparable to the condition found in asphyxiated term human neonates (7, 72).

Doppler sonography and cranial ultrasound proved useful adjuncts in early diagnostic imaging. Magnetic Resonance Imaging (MRI) has been demonstrated to have good diagnostic ability to detect brain injury in cases with HIE, when performed at the end of the first post-natal week of life, and it can support prognosis (65). MRI has also shown value in the animal setting, e.g., due to the correlation of MRI findings with the degree of brain injury in a PA piglet model (71). MRI at 72 h also revealed lesions corresponding to diffuse ischemic infarction in another global hypoxia-ischemia pig model (7).

##### 5.5.1.2. Histopathological examination

In human neonates, PA can cause pathological changes in multiple organs, with HIE representing one of the most severe consequences, and occasionally leading to a multi-organ dysfunction syndrome. The pathological diagnosis of PA is complex, histological markers of tissue hypoxia often overlap with pathological changes due to other causes. The endothelial lesions, represented by swelling, apoptosis, detachment, and loss of the endothelial barrier, are the most relevant pathological changes induced by hypoxia in all organs (69). In the pig model, brain injury after PA is hypoxic-ischemic in nature, and the injury pattern includes selective neuronal ischemic necrosis (7, 62). Additionally, the lungs are commonly affected by PA. Pulmonary hypertension, hemorrhage, and significant coagulopathy are frequent complications (75). The finding of increased hepatic hematopoiesis represents one of the most important markers of chronic tissue hypoxia. Neonates with PA are known to have an increased risk of ischemic heart injury due to decreased cardiac output and decreased coronary perfusion (76). The cardiac injury diagnostic is determined by the presence of high levels of cardiac enzymes; however, the immediate and long-term structural consequences are not well known, since reported histological findings are minimal (77, 78). Therefore, for future PA animal model development, we recommend the analysis of the brain through electrophysiology and neuroimaging, together with the histopathological examination of the brain and other organs such as lungs, heart, liver, and kidneys. With the current survival rate, the sampling time, or strategies to improve postnatal survival should be further considered.

### 5.6. Comparative view on perinatal asphyxia animal models

Numerous models of hypoxia, hypoxia-ischemia, intraventricular hemorrhage, and focal stroke have been developed in rodents and in larger species to mimic the different types of injuries seen in the human newborn (70). Although no animal model is perfectly ideal in terms of capturing the diversity and complexity of human pathology, the investigator must evaluate the strengths and limitations in the context of the research questions (77). Firstly, the maturational pattern of the animal model should be as close as possible to the human newborn, for a better interpretation of the findings. Secondly, the body size at birth should allow the instrumentation (for hemodynamic and physiological measurements), in order to closely monitor the effects of hypoxia and reoxygenation, and the sampling (e.g., blood gas analysis to assess pulmonary gas exchange and acid-base status). This monitoring is crucial in reaching a degree of hypoxia likely to produce significant damage without excessive mortality, and it also helps ensure the reproducibility of the injury (e.g., a similar degree of acidosis with a similar descent over time). Lastly, the type, severity and duration of hypoxic insult are critical to the translational value of the model, which should approximate the clinical picture of PA (79).

The rat pup has been the most commonly used, and thus is probably the best characterized model in HIE research. The newborn rat is altricial (born immature). At birth, its development corresponds to a 24- to 28-week human fetus (79). The most commonly used nonrodent large mammal species to induce HIE in the immature brain are sheep, rabbits, and pigs, species that have a white/gray matter ratio similar to the human brain (70). An overview of the most important criteria in the differentiation of the rodent and non-rodent species for PA/HIE research is presented in Table 4. While impractical as a fetal model due to its large litter size (>10 piglets per litter), the neonatal piglet has been proven to be a valuable model to study HIE. After the rat, the neonatal pig model is the most frequently used. Its development corresponds to the 36- to 38-week human fetus (79). Additionally, concerning drug development research, high homology to the human Phase I drug metabolizing CYP family has already been described in minipigs (63%–84% amino acid identity) (80, 81) and the ontogeny of CYP enzyme activity in the juvenile Göttingen Minipig showed to be comparable to the corresponding age groups in human (19, 21). A comparative overview of the different large animal models developed for PA/HIE, with their strengths and limitations, is provided in Table 5. Therefore, for the aim of our experimental study, the neonatal Göttingen Minipig model was considered more suitable than the rodent model due to its high homology in drug metabolizing enzymes, and its (relatively) larger body size at birth, which allows sampling (i.e., PK) at early stages without hampering the physiology and also facilitates the adaptation of NICU equipment.

**Table 4:** Strengths and limitations of different animal models for * PA (perinatal asphyxia)/HIE (hypoxic ischemic encephalopathy): rodent model versus large animal models.

Animal model for PA/HIE*	Strengths	Limitations
Rodent model	- Postnatal cerebral development analogous to human development in the third trimester. - Litters are easily produced and handled (77).	- Lissencephalic cortex; the CBF^*^ regulation and the white/gray matter ratios is different to the human brain. - Limited behavioral repertory (77). - Rodent brain regions are mature at a different pace, thus making it difficult to adhere to a single postnatal day, as a comprehensive representation of brain development (70). - Rat pups are generally not instrumented to monitor the hypoxia effects on hemodynamic indices, and this can make it difficult to control the degree of hypoxic-ischemic insult (79).
Large animal models	- Gyrencephalic; the white/gray matter ratio is similar to the human brain. - When global hypoxia and hypotension are combined, the results in permanent brain injury, organ failure, post-hypoxic seizures, and abnormal neurology akin to human neonates are documented. - Similar in survival rate. - Among large neonatal animal models, other than nonhuman primates, the (neonatal) pigs are appropriate because their general brain and organ maturation at-term are similar to humans (77).	- High maintenance costs and long-term neurorehabilitation have dramatically limited the use of larger species over the past decade (70). - Ethical reasons (77).

*CBF, cerebral blood flow.

**Table 5:** Comparative overview of the different large animal models used in the study of *PA (perinatal asphyxia)/HIE (hypoxic ischemic encephalopathy), with their strengths and limitations.

Animal model for PA/HIE*	Strengths	Limitations
Nonhuman primate	- Anatomical and physiological similarities to humans (77, 82) due to the phylogenetic proximity (79). - Similar pathological distribution of brain injury (82), allowing the investigation with sophisticated neurological tests (77).	- Other (non-related brain) pathological processes secondary to asphyxia may be more appropriately assessed in other species (82). - In terms of cerebral maturity, nonhuman primates are more precocious than humans and the model's equivalence to the human newborn is unknown (79). - High economic handling costs, in addition to the ethical issues (77).
Sheep	- The instrumentation is feasible due to relatively large body size. - The litter size (one or two offspring) is practical; it is a good model to reproduce the PA conditions prevailing in the womb (79). - Relatively low costs. - Fetal physiology has already been extensively studied in sheep. - Resistance to preterm labor following surgery (82).	- Precocial species; studies are performed during pregnancy to correlate to relevant maturation stages in the human. - Fetal models are complicated by maternal/placenta metabolism, which is not present in the HIE human situation (70). - Different placental flow patterns (cotyledonary placenta, in sheep versus mono-discoidal type, with a single disc, in humans) (82).
Rabbit	- Useful animal model due to the possible application in both, basic research, and clinical applications. - Their medium size greatly facilitates handling with reducing costs (77). - Model for CBF^*^ (83).	- Intrauterine ischemia is induced at approximately 22 days of gestation as equivalent to the preterm, and at the end of gestation, at 29 days, to mimic at-term injury (70).
Dog	- The neonatal beagle is known to have a germinal matrix layer similar to 30- to 32-week human gestational age and thus provides a good model for the developing nervous system research. - Used for the study of regional CBF, glucose, lactate and energy metabolism, autoregulation, maturation of germinal matrix, and drug effects (84).	- Inadequate ventilation, along with clinical risks comparable to the development of respiratory distress syndrome (23).
Pig	- Development similar to (near)term (36- to 38-weeks old) human neonates, with comparable body systems and size at birth (1.5-2 kg). - The large body size at birth allows for sampling at early stages without hampering the physiology and also facilitates the adaptation of NICU^*^ equipment (79). - As the piglet asphyxia model is well-characterized, this opens opportunities for pharmacological interventions and procedures applied in a clinical setting, such as TH (5).	- Anesthesia and surgical trauma stress, limit this model in terms of survival length and number of invasive procedures performed. - Skilled personnel experienced with surgery and anesthesia is the key for these types of experiments (6). - Logistical drawback of getting neonatal piglets in the research facility, when no (pregnant) sows are housed on-site. - Using older piglets (2 or more days of age) risks weakening the clinical relevance of the piglet model, as the model should be akin to the human neonatal metabolism (44).

*CBF, cerebral blood flow; TH, therapeutic hypothermia; NICU, neonatal intensive care unit.

#### 5.6.1. Other pig perinatal asphyxia models

The most critical step in developing this model was generating a standardized hypoxic insult, as the primary goal was to induce a moderate to severe insult while ensuring animal survival. Before setting up this protocol, we analyzed previously performed hypoxia pig models for a comparative methodology and knowledge of limitations. In view of the PA simulation, Cheung et al. (6), described an experimental model represented by surgically instrumented newborn piglets, involved in a non-survival experiment, that allows for mechanical ventilation, arterial and central venous access and the placement of catheters and flow probes for the continuous monitoring of intra-vascular pressure and blood flow, across different arteries. In this protocol, severe hypoxemia was achieved by decreasing the inspired oxygen concentration to 10%–15% and by increasing the concentration of inhaled nitrogen gas, for 2 h, aiming for 30%–40% SpO_2_ (6). A similar piglet model for PA was proposed by O'Brien et al. (62), via whole-body hypoxia-asphyxia, induced by decreasing the inhaled oxygen to 10%, for 45 min in combination with the occlusion of the ETT, for seven min. Global hypoxia-ischemia was demonstrated in a HIE piglet model, by Kyng et al. (7), combining ischemia, through hypotension, with low FiO_2_ via nitrogen gas, and a flat trace aEEG, indicative of cerebral hypoxia. Survival was promoted by adjusting oxygenation according to the aEEG response and blood pressure. A different approach for inducing transient hypoxia-ischemia was presented by Ezzati et al. (8), by remote occlusion of both common carotid arteries, using inflatable vascular occluders, at the level of the fourth cervical vertebra, with low FiO_2_ of 0.09. In our study, the monitoring of the laboratory clues for acidosis, via blood gas analysis, and the perfusion and cardiac function, via ECG and blood pressure, allowed for adjustments according to each piglet´s tolerance to hypoxia, thus ensuring a high survival rate.

## 6. Conclusion

In this study, a neonatal Göttingen Minipig model for dose precision in PA was developed. We showed that endotracheal intubation, vascular access, anesthesia, and mechanical ventilation are feasible in these very small animals. Therefore, this is useful information for scientists using the neonatal Göttingen Minipig for investigating disease conditions or for drug efficacy and safety testing, since it is the most commonly used pig strain in nonclinical drug development. This model will be further used to assess the impact of systemic hypoxia and TH on drug disposition resulting in better drug dosing and drug exposure in human asphyxiated neonates. Besides the success of several challenging techniques achieved in this neonatal Göttingen Minipig model, the current model also shows limitations such as the short survival duration (24 h), which results in several consequences (e.g., no rewarming phase and limited PK profile of the drugs studied). Furthermore, additional diagnostic tools would be useful for the evaluation of HIE in this model.

## Data Availability

The original contributions presented in the study are included in the article/**Supplementary Material**, further inquiries can be directed to the corresponding author.

## References

[1] Helke KL Swindle MM. Animal models of toxicology testing: the role of pigs. Expert Opin Drug Metab Toxicol. (2013) 9(2):127–39. 10.1517/17425255.2013.739607 23216131

[2] Singh VK Thrall KD Hauer-Jensen M. Minipigs as models in drug discovery. Expert Opin Drug Discov. (2016) 11(12):1131–4. 10.1080/17460441.2016.1223039 27546211

[3] European Medicines Agency. Committee for Medicinal Products for Human Use, ICH guideline S11 on nonclinical safety testing in support of development of paediatric pharmaceuticals, 31 March 2020, EMA/CHMP/ICH/616110/2018. https://www.ema.europa.eu/en/ich-guideline-s11-nonclinical-safety-testing-support-development-paediatric-pharmaceuticals-step-5

[4] EFSA Scientific Committee, Hardy A Benford D Halldorsson T Jeger MJ Knutsen HK Guidance on the risk assessment of substances present in food intended for infants below 16 weeks of age. EFSA J. (2017) 15(5):e04849. 10.2903/j.efsa.2017.4849 32625502 PMC7010120

[5] Ayuso M Buyssens L Stroe M Valenzuela A Allegaert K Smits A The neonatal and juvenile pig in pediatric drug discovery and development. Pharmaceutics. (2021) 13(1):44. 10.3390/pharmaceutics13010044 PMC7823749 33396805

[6] Cheung P-Y Gill RS Bigam DL. A swine model of neonatal asphyxia. J Vis Exp. (2011) 56:3166. 10.3791/3166 PMC3227176 22006174

[7] Kyng KJ Skajaa T Kerrn-Jespersen S Andreassen CS Bennedsgaard K Henriksen TB. A piglet model of neonatal hypoxic-ischemic encephalopathy. J Vis Exp. (2015) 99:e52454. 10.3791/52454 PMC4542803 26068784

[8] Ezzati M Broad K Kawano G Faulkner S Hassell J Fleiss B Pharmacokinetics of dexmedetomidine combined with therapeutic hypothermia in a piglet asphyxia model. Acta Anaesthesiol Scand. (2014) 58(6):733–42. 10.1111/aas.12318 24724965 PMC4171780

[9] Smits A Annaert P Van Cruchten S Allegaert K. A physiology-based pharmacokinetic framework to support drug development and dose precision during therapeutic hypothermia in neonates. Front Pharmacol. (2020) 11:587. 10.3389/fphar.2020.00587 32477113 PMC7237643

[10] Pearson N Boiczyk GM Kote VB Sundaramurthy A Subramaniam DR Rubio JE A strain rate-dependent constitutive model for Göttingen Minipig cerebral arteries. J Biomech Eng. (2022) 144(8):081007. 10.1115/1.4053796 35147172

[11] Musskopf ML Finger Stadler A Wikesjö UME Susin C. The minipig intraoral dental implant model: a systematic review and meta-analysis. PLoS One. (2022) 17(2):e0264475. 10.1371/journal.pone.0264475 35226690 PMC8884544

[12] Larsen MO Rolin B. Use of the Göttingen Minipig as a model of diabetes, with special focus on type 1 diabetes research. ILAR J. (2004) 45(3):303–13. 10.1093/ilar.45.3.303 15229377

[13] Roh J Hill JA Singh A Valero-Muñoz M Sam F. Heart failure with preserved ejection fraction: heterogeneous syndrome, diverse preclinical models. Circ Res. (2022) 130(12):1906–25. 10.1161/CIRCRESAHA.122.320257 35679364 PMC10035274

[14] Eirefelt S Stahlhut M Svitacheva N Carnerup MA Da Rosa JMC Ewald DA Characterization of a novel non-steroidal glucocorticoid receptor agonist optimized for topical treatment. Sci Rep. (2022) 12(1):1501. 10.1038/s41598-022-05471-w 35087193 PMC8795149

[15] Eisler W Baur J-O Held M Rahmanian-Schwarz A Daigeler A Denzinger M. Assessment of two commonly used dermal regeneration templates in a swine model without skin grafting. Appl Sci. (2022) 12(6):3205. 10.3390/app12063205

[16] Stricker-Krongrad A Shoemake CR Pereira ME Gad SC Brocksmith D Bouchard GF. Miniature swine breeds in toxicology and drug safety assessments: what to expect during clinical and pathology evaluations. Toxicol Pathol. (2015) 44(3):421–7. 10.1177/0192623315613337 26656239

[17] Bode G Clausing P Gervais F Loegsted J Luft J Nogues V The utility of the minipig as an animal model in regulatory toxicology. J Pharmacol Toxicol Methods. (2010) 62(3):196–220. 10.1016/j.vascn.2010.05.009 20685310

[18] Valenzuela A Tardiveau C Ayuso M Buyssens L Bars C Van Ginneken C Safety testing of an antisense oligonucleotide intended for pediatric indications in the juvenile Göttingen Minipig, including an evaluation of the ontogeny of key nucleases. Pharmaceutics. (2021) 13(9):1442. 10.3390/pharmaceutics13091442 34575518 PMC8470776

[19] Buyssens L De Clerck L Schelstraete W Dhaenens M Deforce D Ayuso M Hepatic cytochrome P450 abundance and activity in the developing and adult Göttingen Minipig: pivotal data for PBPK modeling. Front Pharmacol. (2021) 12:665644. 10.3389/fphar.2021.665644 33935788 PMC8082684

[20] Van Peer E De Bock L Boussery K Van Bocxlaer J Casteleyn C Van Ginneken C Age-related differences in CYP3A abundance and activity in the liver of the Göttingen Minipig. Basic Clin Pharmacol Toxicol. (2015) 117(5):350–7. 10.1111/bcpt.12410 25892190

[21] Van Peer E Jacobs F Snoeys J Van Houdt J Pijpers I Casteleyn C In vitro phase I-and phase II-drug metabolism in the liver of juvenile and adult Göttingen Minipigs. Pharm Res. (2017) 34(4):750–64. 10.1007/s11095-017-2101-y 28097507

[22] Grimm KA Lamont LA Tranquilli WJ Greene SA Robertson SA. Veterinary anesthesia and analgesia. Chichester, UK: John Wiley & Sons (2015).

[23] Duke-Novakovski T Vries MD Seymour C. BSAVA manual of canine and feline anaesthesia and analgesia. Gloucester, UK: British Small Animal Veterinary Association (2016).

[24] Plumb D. Plumb’s vet drug handbook: Desk edition. Ames, IA: Blackwell (2008).

[25] Galinkin JL Kurth CD Shi H Priestley MA Loepke AW Adamson PC. The plasma pharmacokinetics and cerebral spinal fluid penetration of intravenous topiramate in newborn pigs. Biopharm Drug Dispos. (2004) 25(6):265–71. 10.1002/bdd.408 15334626

[26] Clark AM Kriel RL Leppik IE Marino SE Mishra U Brundage RC Intravenous topiramate: comparison of pharmacokinetics and safety with the oral formulation in healthy volunteers. Epilepsia. (2013) 54(6):1099–105. 10.1111/epi.12134 23506041

[27] Clark AM Kriel RL Leppik IE White JR Henry TR Brundage RC Intravenous topiramate: safety and pharmacokinetics following a single dose in patients with epilepsy or migraines taking oral topiramate. Epilepsia. (2013) 54(6):1106–11. 10.1111/epi.12165 23586686

[28] Vuu I Coles LD Maglalang P Leppik IE Worrell G Crepeau D Intravenous topiramate: pharmacokinetics in dogs with naturally occurring epilepsy. Front Vet Sci. (2016) 3: 10.3389/fvets.2016.00107 27995128 PMC5136567

[29] Villanueva-García D Mota-Rojas D Martinez-Burnes J Olmos-Hernandez A Mora-Medina P Salmerón C Hypothermia in newly born piglets: mechanisms of thermoregulation and pathophysiology of death. J Anim Behav Biometeorol. (2020) 9:2101–9. 10.31893/jabb.21001

[30] Kumar A Mann H Remmel R. Pharmacokinetics of tiletamine and zolazepam (Telazol®) in anesthetized pigs. J Vet Pharmacol Ther. (2006) 29(6):587–9. 10.1111/j.1365-2885.2006.00798.x 17083465

[31] Bigby SE Carter JE Bauquier S Beths T. The use of alfaxalone for premedication, induction and maintenance of anaesthesia in pigs: a pilot study. Vet Anaesth Analg. (2017) 44(4):905–9. 10.1016/j.vaa.2016.06.008 28716685

[32] Sangild PT Thymann T Schmidt M Stoll B Burrin DG Buddington RK. Invited review: the preterm pig as a model in pediatric gastroenterology. J Anim Sci. (2013) 91(10):4713–29. 10.2527/jas.2013-6359 23942716 PMC3984402

[33] Burrin D Stoll B Jiang R Petersen Y Elnif J Buddington R GLP-2 stimulates intestinal growth in premature TPN-fed pigs by suppressing proteolysis and apoptosis. Am J Physiol Gastrointest Liver Physiol. (2000) 279(6):G1249–G56. 10.1152/ajpgi.2000.279.6.G1249 11093948

[34] Zeltner A. Catheters for vascular access and the Göttingen Minipig. Vol. 3. Dalmose, Denmark: Göttingen Minipigs Magazine (2013).

[35] Seldinger SI. Catheter replacement of the needle in percutaneous arteriography: a new technique. Acta Radiol. (1953) 39(5):368–76. 10.3109/00016925309136722 13057644

[36] Song IK Kim EH Lee JH Jang YE Kim HS Kim JT. Seldinger vs modified seldinger techniques for ultrasound-guided central venous catheterisation in neonates: a randomised controlled trial. Br J Anaesth. (2018) 121(6):1332–7. 10.1016/j.bja.2018.08.008 30442261

[37] Furbeyre H Labussiere E. A minimally invasive catheterization of the external jugular vein in suckling piglets using ultrasound guidance. PLoS One. (2020) 15(10):e0241444. 10.1371/journal.pone.0241444 33112934 PMC7592786

[38] Framstad T Sjaastad O Aass RA. Bleeding and intravenous techniques in pigs (2004). https://norecopa.no/education-training/other-teaching-materials/pig-bleeding/, web page updated on 19 December 2022, accessed on 25 April 2023

[39] Snook CS. Use of the subcutaneous abdominal vein for blood sampling and intravenous catheterization in potbellied pigs. J Am Vet Med Assoc. (2001) 219(6):809–10. 10.2460/javma.2001.219.809 11561659

[40] Benoit A Dailey R. Catheterization of the caudal vena cava via the lateral saphenous vein in the ewe, cow, and gilt: an alternative to utero-ovarian and medial coccygeal vein catheters. J Anim Sci. (1991) 69(7):2971–9. 10.2527/1991.6972971x 1885407

[41] Gasthuys F Pollet L Simoens P Lauwers H De Laey J. Anaesthesia for fluorescein angiography of the ocular fundus in the miniature pig. Vet Res Commun. (1990) 14(5):393–402. 10.1007/BF00343217 2247945

[42] Virolainen JV Love RJ Tast A Peltoniemi OA. Plasma progesterone concentration depends on sampling site in pigs. Anim Reprod Sci. (2005) 86(3-4):305–16. 10.1016/j.anireprosci.2004.07.004 15766808

[43] National Institute for Health and Care Excellence (NICE). Interventional procedures guidance [IPG347]. Therapeutic hypothermia with intracorporeal temperature monitoring for hypoxic perinatal brain injury (2010). Published on 26 May 2010, accessed on 25 April 2023

[44] Whitaker EE Zheng CZ Bissonnette B Miller AD Koppert TL Tobias JD Use of a piglet model for the study of anesthetic-induced developmental neurotoxicity (AIDN): a translational neuroscience approach. J Vis Exp. (2017) 124:55193. 10.3791/55193. PMC5608378 28654034

[45] Theisen M Maas M Hartlage M Ploner F Niehues S Van Aken H Ventral recumbency is crucial for fast and safe orotracheal intubation in laboratory swine. Lab Anim. (2008) 43:96–101. 10.1258/la.2008.008044 19015175

[46] Steinbacher R von Ritgen S Moens YPS. Laryngeal perforation during a standard intubation procedure in a pig. Lab Anim. (2012) 46(3):261–3. 10.1258/la.2012.012032 22723649

[47] Hodgkinson O. Practical sedation and anaesthesia in pigs. In Pract. (2007) 29(1):34–9. 10.1136/inpract.29.1.34

[48] Ahluwalia J Morley C Wahle HG. Volume guarantee new approaches in volume controlled ventilation for neonates. Lubeck: Drager Medizintechnik GmbH (2014).

[49] Clifton-Koeppel R. Endotracheal tube suctioning in the newborn: a review of the literature. Newborn Infant Nurs Rev. (2006) 6(2):94–9. 10.1053/j.nainr.2006.03.006

[50] Li C Ren Q Li X Han H Peng M Xie K Effect of sigh in lateral position on postoperative atelectasis in adults assessed by lung ultrasound: a randomized, controlled trial. BMC Anesthesiol. (2022) 22(1):215. 10.1186/s12871-022-01748-9 35820814 PMC9275275

[51] Clutton RE Reed F Eddleston M Hulse E. Prolonged anaesthesia in minipigs. Prolong Anaesth Minipigs. (2013):11–5.

[52] Holte K Ersdal H Eilevstjønn J Gomo Ø Klingenberg C Thallinger M Positive end-expiratory pressure in newborn resuscitation around term: a randomized controlled trial. Pediatrics. (2020) 146(4):e20200494. 10.1542/peds.2020-0494 32917847

[53] Sam Wallis CF. Neonatal ventilation – basics of mechanical ventilation. Bradford Teaching Hospitals NHS Foundation Trust [Internet]. (2022). Available at: https://www.bradfordhospitals.nhs.uk/wp-content/uploads/2020/07/Neonatal-Ventilation.pdf.

[54] Kuchnicka K Maciejewski D. Ventilator-associated lung injury. Anaesthesiol Intensive Ther. (2013) 45(3):164–70. 10.5603/AIT.2013.0034 24092514

[55] D'Andrea V Prontera G Rubortone SA Pezza L Pinna G Barone G Umbilical venous catheter update: a narrative review including ultrasound and training. Front Pediatr. (2021) 9:774705. 10.3389/fped.2021.774705 35174113 PMC8841780

[56] Hermansen MC Hermansen MG. Intravascular catheter complications in the neonatal intensive care unit. Clin Perinatol. (2005) 32(1):141–56. 10.1016/j.clp.2004.11.005 15777826

[57] Yu I Lin J Wu J Yen H Lee S Yang T. Reevaluation of the necessity of iron injection to newborn piglets. Asian Australas J Anim Sci. (2002) 15(1):79–83. 10.5713/ajas.2002.79

[58] Chevalier TB Monegue HJ Lindemann MD. Effects of iron dosage administered to newborn piglets on hematological measures, preweaning and postweaning growth performance, and postweaning tissue mineral content. J Swine Health Prod. (2021) 29(4):189–99.

[59] Meyer A Paroni F Günther K Dharmadhikari G Ahrens W Kelm S Evaluation of existing methods for human blood mRNA isolation and analysis for large studies. PLoS One. (2016) 11(8):e0161778. 10.1371/journal.pone.0161778 27575051 PMC5004844

[60] Brodbelt D. Perioperative mortality in small animal anaesthesia. Vet J. (2009) 182(2):152–61. 10.1016/j.tvjl.2008.06.011 18658000

[61] Larson AC Jamrogowicz JL Kulikowicz E Wang B Yang ZJ Shaffner DH Cerebrovascular autoregulation after rewarming from hypothermia in a neonatal swine model of asphyxic brain injury. J Appl Physiol. (2013) 115(10):1433–42. 10.1152/japplphysiol.00238.2013 24009008 PMC3841826

[62] O’Brien CE Santos PT Kulikowicz E Reyes M Koehler RC Martin LJ Hypoxia-ischemia and hypothermia independently and interactively affect neuronal pathology in neonatal piglets with short-term recovery. Dev Neurosci. (2019) 41(1-2):17–33. 10.1159/000496602 31108487 PMC6732227

[63] Carlsen MF Christoffersen BØ Lindgaard R Pedersen HD Olsen LH. Implantation of telemetric blood pressure transmitters in Göttingen Minipigs: validation of 24-h systemic blood pressure and heart rate monitoring and influence of anaesthesia. J Pharmacol Toxicol Methods. (2022) 115:107168. 10.1016/j.vascn.2022.107168 35315338

[64] Azzopardi D Brocklehurst P Edwards D Halliday H Levene M Thoresen M The TOBY study. Whole body hypothermia for the treatment of perinatal asphyxial encephalopathy: a randomised controlled trial. BMC Pediatr. (2008) 8(1):17. 10.1186/1471-2431-8-17 18447921 PMC2409316

[65] O'Dea M Sweetman D Bonifacio SL El-Dib M Austin T Molloy EJ. Management of multi organ dysfunction in neonatal encephalopathy. Front Pediatr. (2020) 8:239. 10.3389/fped.2020.00239 32500050 PMC7243796

[66] Moshiro R Mdoe P Perlman JM. A global view of neonatal asphyxia and resuscitation. Front Pediatr. (2019) 7:489. 10.3389/fped.2019.00489 31850287 PMC6902004

[67] Gomella TL Cunningham MD Eyal FG Tuttle DJ. Perinatal asphyxia. In: Gomella T, Eyal FG, Bany-Mohammed F, editors, Neonatology: Management, procedures, on-call problems, diseases, and drugs. 8ed. New York, NY: McGraw-Hill Education (2020). Accessed April 25, 2023. https://accesspediatrics.mhmedical.com/content.aspx?bookid=2762&sectionid=234452100

[68] Hines RN. The ontogeny of drug metabolism enzymes and implications for adverse drug events. Pharmacol Ther. (2008) 118(2):250–67. 10.1016/j.pharmthera.2008.02.005 18406467

[69] Gerosa C Fanni D Puddu M Locci G Obinu E Fanos V Histological markers of neonatal asphyxia: the relevant role of vascular changes. J Pediatr Neonat Individual Med. (2014) 3(2):e030275. 10.7363/030275

[70] Mallard C Vexler ZS. Modeling ischemia in the immature brain. Stroke. (2015) 46(10):3006–11. 10.1161/STROKEAHA.115.007776 26272384 PMC4589478

[71] Robertson NJ Thayyil S Cady EB Raivich G. Magnetic resonance spectroscopy biomarkers in term perinatal asphyxial encephalopathy: from neuropathological correlates to future clinical applications. Curr Pediatr Rev. (2014) 10(1):37–47. 10.2174/157339631001140408120613 25055862

[72] Liu X Tooley J Løberg EM Suleiman MS Thoresen M. Immediate hypothermia reduces cardiac troponin I after hypoxic-ischemic encephalopathy in newborn pigs. Pediatr Res. (2011) 70(4):352–6. 10.1203/PDR.0b013e31822941ee 21691250 PMC3173864

[73] Gacio S. Amplitude-integrated electroencephalography for neonatal seizure detection. An electrophysiological point of view. Arq Neuropsiquiatr. (2019) 77(2):122–30. 10.1590/0004-282x20180150 30810597

[74] Shankaran S Pappas A McDonald SA Laptook AR Bara R Ehrenkranz RA Predictive value of an early amplitude integrated electroencephalogram and neurologic examination. Pediatrics. (2011) 128(1):e112–20. 10.1542/peds.2010-2036 21669899 PMC3124102

[75] Forman KR Diab Y Wong EC Baumgart S Luban NL Massaro AN. Coagulopathy in newborns with hypoxic ischemic encephalopathy (HIE) treated with therapeutic hypothermia: a retrospective case-control study. BMC Pediatr. (2014) 14:277. 10.1186/1471-2431-14-277 25367591 PMC4289197

[76] Sehgal A Wong F Mehta S. Reduced cardiac output and its correlation with coronary blood flow and troponin in asphyxiated infants treated with therapeutic hypothermia. Eur J Pediatr. (2012) 171(10):1511–7. 10.1007/s00431-012-1764-y 22669637

[77] Mota-Rojas D Villanueva-García D Solimano A Muns R Ibarra-Ríos D Mota-Reyes A. Pathophysiology of perinatal asphyxia in humans and animal models. Biomedicines. (2022) 10(2):347. 10.3390/biomedicines10020347 35203556 PMC8961792

[78] LaRosa DA Ellery SJ Walker DW Dickinson H. Understanding the full spectrum of organ injury following intrapartum asphyxia. Front Pediatr. (2017) 5:16. 10.3389/fped.2017.00016 28261573 PMC5313537

[79] Chapados I Cheung PY. Not all models are created equal: animal models to study hypoxic-ischemic encephalopathy of the newborn. Commentary on Gelfand SL et al.: a new model of oxidative stress in rat pups (Neonatology 2008;94:293-299). Neonatology. (2008) 94(4):300–3. 10.1159/000151650 18784427

[80] Puccinelli E Gervasi P Longo V. Xenobiotic metabolizing cytochrome P450 in pig, a promising animal model. Curr Drug Metab. (2011) 12:507–25. 10.2174/138920011795713698 21476973

[81] Heckel T Schmucki R Berrera M Ringshandl S Badi L Steiner G Functional analysis and transcriptional output of the Göttingen Minipig genome. BMC Genom. (2015) 16:932. 10.1186/s12864-015-2119-7 PMC4647470 26573612

[82] Painter MJ. Animal models of perinatal asphyxia: contributions, contradictions, clinical relevance. Semin Pediatr Neurol. (1995) 2(1):37–56. 10.1016/S1071-9091(05)80004-X 9422233

[83] Raju TN. Some animal models for the study of perinatal asphyxia. Biol Neonate. (1992) 62(4):202–14. 10.1159/000243873 1420619

[84] Ment LR Stewart WB Gore JC Duncan CC. Beagle puppy model of perinatal asphyxia: alterations in cerebral blood flow and metabolism. Pediatr Neurol. (1988) 4(2):98–104. 10.1016/0887-8994(88)90048-3 3149477

