# Supplementary material for: Development of a neonatal Göttingen Minipig model for dose precision in perinatal asphyxia: technical opportunities, challenges, and potential further steps
Source: Front Pediatr. 2023 May 4;11:1163100. doi: 10.3389/fped.2023.1163100 (PMC10195037; doi:10.3389/fped.2023.1163100)
Supplement: Supplementary file 1 [file Table1.docx]

Supplementary Material

Development of a neonatal Göttingen Minipig model for dose precision in perinatal asphyxia: technical opportunities, challenges, and potential further steps

Marina-Stefania Stroe^1^, Lieselotte Van Bockstal^1^, Allan Valenzuela^1^, Miriam Ayuso^1^, Karen Leys^2^, Pieter Annaert^2,3^, Sebastien Carpentier^4^, Anne Smits^5,6^, Karel Allegaert^6,7,8^, Adrian Zeltner^9^, Antonius Mulder^10^, Chris Van Ginneken^1^, Steven Van Cruchten^1*^

*** Correspondence:**Steven Van Cruchten

[steven.vancruchten@uantwerpen.be](mailto:steven.vancruchten@uantwerpen.be)

## Supplementary blood gas analysis parameters, glucose, and body temperatures for the experimental study groups (control (C), therapeutic hypothermia (TH), hypoxia (H), hypoxia and therapeutic hypothermia (H+TH)) in neonatal Göttingen Minipigs. Blood gas analysis was performed with i-STAT® Alinity V and the Accutrend® Plus System (Roche), using central venous blood. The assessment was performed at the end of the experiment (i.e., after 24h) for C and TH groups. For H, and H+TH groups, the parameters were determined during, at the end of the hypoxic insult and at the end of the 24h TH. * PCO2 (partial pressure of carbon dioxide); PO2 (partial pressure of oxygen); HCO3 (bicarbonate); BE (base excess); sO2 (oxygen saturation); TCO2 (total carbon dioxide); T °C (temperature at the moment of collection).

## Control group (C) :

| Subject | pH | PCO2 (kPa) | PO2 (kPa) | HCO3 (mmol/l) | BE (mmol/l) | sO2 (mmol/l) | TCO2 (mmol/l) | Lactate (mmol/l) | Glucose (mg/dl) | T (°C) |
| --- | --- | --- | --- | --- | --- | --- | --- | --- | --- | --- |
| C1 | 7.391 | 6.02 | 5.9 | 27.4 | 2 | 79 | 29 | 1.34 | 75 | 37.6 |
| C2 | 7.145 | 12.19 | 5.8 | 31.5 | 3 | 62 | 34 | 1.73 | 133 | 37.7 |
| C3 | 7.36 | 9.4 | 4.9 | 39.8 | 14 | 65 | 42 | 1.18 | 90 | 37.2 |
| C4 | 7.079 | 14.46 | 3.8 | 32.1 | 2 | 31 | 35 | 0.3 | 96 | 37.8 |
| C5 | 7.293 | 8.5 | 5.3 | 30.9 | 4 | 67 | 33 | 0.57 | 116 | 38.4 |
| C6 | 7.336 | 8.4 | 7.1 | 33.7 | 8 | 84 | 36 | 0.72 | 74 | 37.5 |

## Therapeutic hypothermia group (TH) :

| Subject | pH | PCO2 (kPa) | PO2 (kPa) | HCO3 (mmol/l) | BE (mmol/l) | sO2 (mmol/l) | TCO2 (mmol/l) | Lactate (mmol/l) | Glucose (mg/dl) | T (°C) |
| --- | --- | --- | --- | --- | --- | --- | --- | --- | --- | --- |
| TH1 | 7.257 | 9.64 | 5.6 | 32.2 | 5 | 68 | 34 | 1 | 30 | 34.7 |
| TH2 | 7.086 | 13.16 | 9 | 29.7 | 0 | 83 | 33 | 0.3 | 47 | 35.2 |
| TH3 | 7.091 | 15.59 | 5.5 | 35.6 | 6 | 54 | 39 | 2.36 | 122 | 34.8 |
| TH4 | 6.888 | 17.33 | 7.6 | - | - | - | - | 0.3 | 75 | 32.7 |
| TH5 | 7.202 | 13.77 | 5.8 | 40.5 | 13 | 64 | 44 | 0.85 | 78 | 33.5 |
| TH6 | 7.083 | 17.33 | 8.6 | - | - | - | - | 0.3 | 87 | 33.7 |

## Hypoxia group (H):

During the hypoxic insult, blood gas analysis was performed at 30 min and approximately one hour after start. Once the blood lactate increased to the threshold of 8.7-10.7 mmol/l and pH decreased from the individual baseline determined before the insult, hypoxia was terminated, at approximately one hour. When the low oxygen gas mix ventilation was insufficient to achieve the targeted blood gas parameters, occlusion of the ETT was performed for seven min, thus adding hypercapnia to the hypoxia. * Bold style: Lactate measurements using the Accutrend® Plus System with the Accutrend® Lactate Strips (Roche). This monitoring system allows lactate determination more rapidly (90 seconds), using 1 drop of central venous blood.

## Hypoxia and therapeutic hypothermia group (H+TH):

Note 1: For H+TH3 the systemic hypoxic insult and 24h survival was achieved. However, at the end of the experiment, misplacement of the epigastric catheter was noticed. Consequently, this subject was excluded from the pharmacokinetic analysis. Since this animal was excluded due to a technical and not a physiological issue, its blood gas analysis parameters were still considered for the hypoxia statistical analysis.

# Trend lines of the pharmacodynamic parameters and body temperatures at the moment of collection during the systemic hypoxic insult, in hypoxia (A), and hypoxia + therapeutic hypothermia (H+TH) (B) groups in neonatal Göttingen Minipigs. * HR (heart rate), bpm (beats per minute); SpO2 (fraction of oxygen-saturated hemoglobin); EtCO2 (end-tidal carbon dioxide).


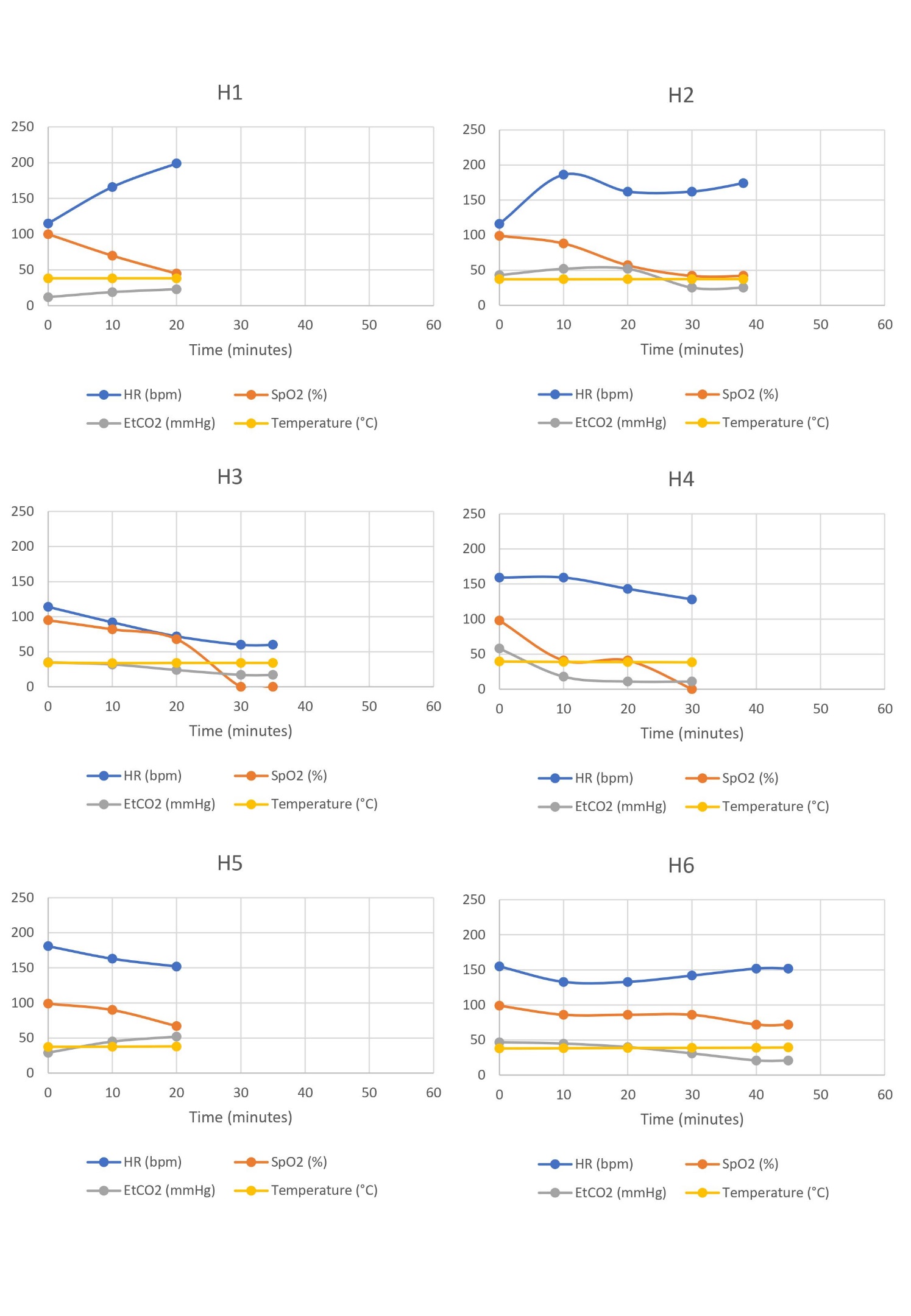


**A.**


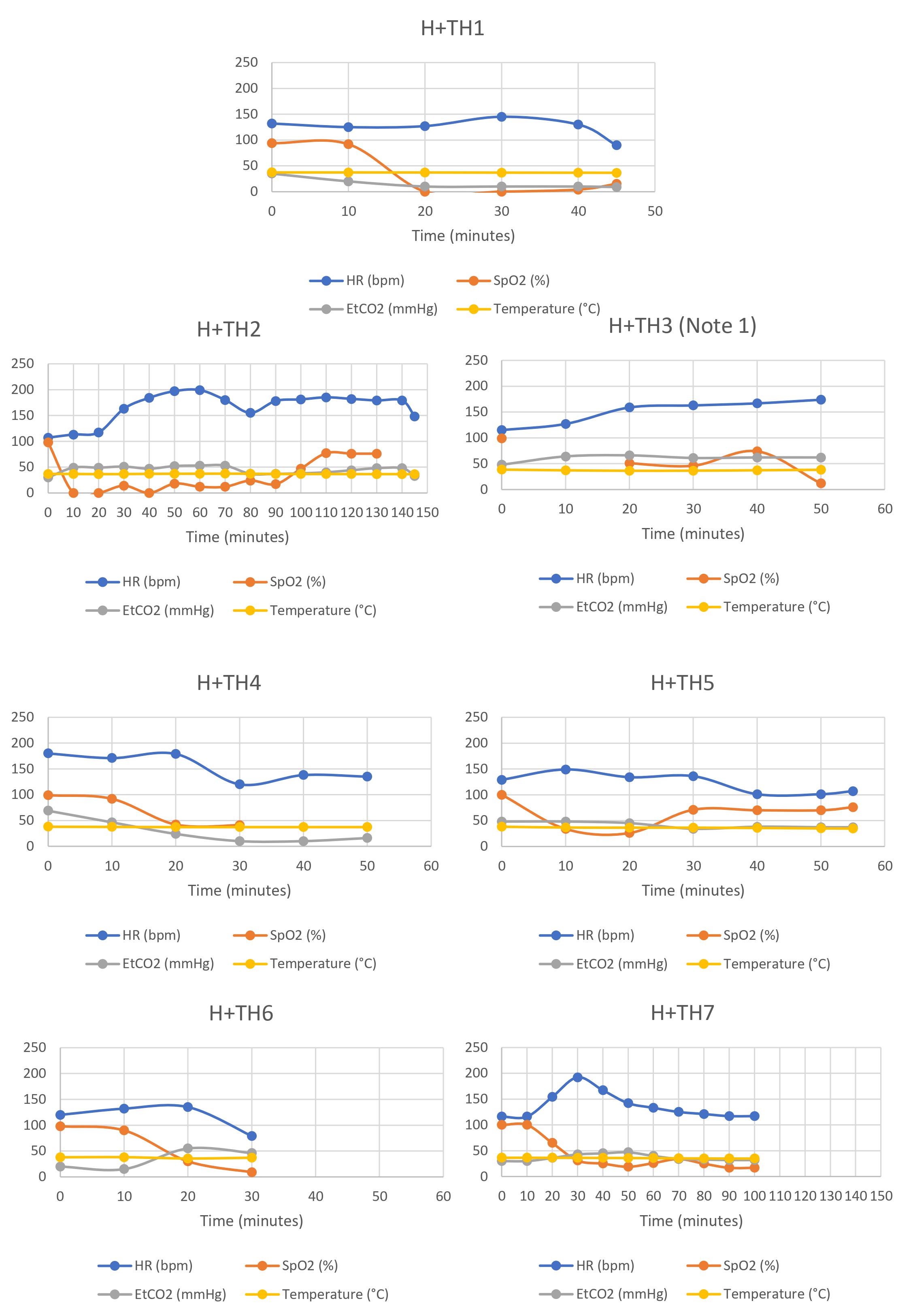


**B.**
